# CFD microscale modelling of flow behavior in different parts of a rotating packed bed

**DOI:** 10.1038/s41598-023-49905-5

**Published:** 2023-12-16

**Authors:** Ahmed M. Alatyar, Abdallah S. Berrouk, Mohamed S. AlShehhi

**Affiliations:** 1 Mechanical Engineering Department, Khalifa University of Science and Technology, P.O. Box 127788, Abu Dhabi, United Arab Emirates; 2 Center for Catalysis and Separation (CeCas), Khalifa University of Science and Technology, P.O. Box 127788, Abu Dhabi, United Arab Emirates; 3 Mechanical Power Engineering Department, Faculty of Engineering, Tanta University, P.O. Box 31521, Tanta, Egypt

**Keywords:** Energy science and technology, Engineering, Mathematics and computing

## Abstract

Process intensification (PI) is playing a key role in alleviating the challenge of reducing carbon footprint of many chemical processes and bringing down their development costs. Over the years, many PI technologies have been investigated with rotating packed bed (RPB) technology receiving much of the attention for its potential of significant intensification in terms of capital expenditure, operating costs, and hardware size. In this study, microscale CFD simulations of a rotating packed bed were conducted, and the results were validated with experimental data. The results show the strong relation between the reverse flow at the packing outer periphery and the gas maldistribution factor. The latter is mainly caused by the accelerating flow in the outer cavity. Inside the wire mesh packing, the gas flow is found to be almost fully uniform for nearly half of the total packing depth. Also, turbulent kinetic energy (TKE) levels at the packing outer edge are strongly linked to the slip tangential velocity component, while at its inner edge, they depend mainly on the radial packing velocity. The so-called gas end effect zone is detected by observing the TKE profiles near the packing outer edge. The latter accounts for less than 10% of the total packing depth. The validity of the widely used porous media model in RPBs’ packing for both radial and tangential directions is confirmed by the obtained results, but this excludes the packing inner and outer edges. In the inner cavity region, gas exhibits two distinctive behaviors and transits from free vortex flow to swirling flow as the flow becomes close to the vortex core. As a result of this transition, the increase in shear stress accelerates the decrease in the gas tangential velocity in the vortex core and help speed up the favorable pressure gradient and flow establishment beyond the vortex core.

## Introduction

Process intensification (PI) is an engineering concept developed to improve physical and chemical processes’ compactness, energy efficiency, safety, and carbon footprint^1,2^. This is often achieved by reducing the size of the process hardware and/or by integrating more than one process function into a single process. Among the different PI technologies, high gravity field technology (HiGee) has been used for enhancing heat and momentum transfer in gas–liquid contactors for the last four decades^3^. It substitutes terrestrial gravity, used by conventional distillation columns, with an intense centrifugal acceleration field. The latter helps increase the mass transfer capacity in a reduced-size design, which eventually decreases significantly both capital and operating costs for the same throughput. For instance, a conventional packed bed absorber of 14-m height and 4.4-m diameter can achieve an equivalent height of transfer units (HTU) of around 3.4 m when used for solvent absorption of CO_2_. This HTU can be reduced drastically to 0.27 m if replaced with a 0.025-m height and 0.398-m diameter rotating absorber^4^. Similar findings are also reported by Lin and Liu^5^ and Cheng and Tan^6^. The use of centrifugal acceleration concept for separation stands behind the design of many process- intensifying gas–liquid contactors such as spinning disk reactors (SDR), rotating spiral contactors (RSC), and rotating packed bed reactors (RPBR). The latter has been the most successful HiGee type of reactors in achieving several-order-of-magnitude increase of mass transfer rates for many types of processes^7^. In RPB, solvent enters the hub region of the packing and flows radially outward under the centrifugal field. Gas, on the other hand, enters the packing through the outer edge, where it meets the liquid solvent in a counter current mode before it exits from the inner edge. As centrifugal force can reach up to 100 times the one due to terrestrial gravity, liquid solvent breaks up into thin liquid filaments and further into very small droplets. This yields a significant increase of gas–liquid interfacial area and hence mass transfer rates. The latter is also boosted by the rapid mixing that is due to the chaotic behavior of liquid flow through the packing^8^. To tap into the huge process-intensifying potential of RPBs, different measurement techniques and variants of CFD models have been used by numerous researchers to thoroughly study the hydrodynamic and mass transfer performance of these devices. A body of experimental work has been devoted to investigate the overall performance parameters of RPBs such as dry and wet pressure drops^9^, liquid hold up^10^, turbulent kinetic energy^11^, and effective interfacial area^12^. The experimental investigations have also reported many challenges that have affected the accuracy of the measurements and that are mainly related to the complexity of the packing structures and the difficulty of synchronization with the bed rotation. These difficulties have been alleviated to a certain extent by resorting to some modified (hence expensive) experimental techniques such as Particle Image Velocimetry (PIV)^11^ and Tomography^13^. Also, Computational Fluid Dynamics (CFD) can play an important role in simulation and visualization of flow behavior within RPBs under different designs and different operating conditions. These CFD models can be categorized into macroscale models (Porous media models) and microscale models. The former has been extensively used to study flow behavior within RPBs since they use porous media model for the packing part of RPBs^14–19^.

The latter does not require very fine mesh to capture the flow within RPBs' packing and hence the flow is not resolved in all its details within the packing structure. The Darcy- Forchheimer equation^20^ with Ergun coefficients^21^ has been widely used in this type of modelling by introducing the so-called inertial and viscous resistance forces to mimic the packing shear forces. The latter models, microscale models, should in principle better predict the hydrodynamics within RPBs. Unlike macroscale modelling, they do not need modeling of drag forces between fluid and packing since they resolve the flow in all its details in every part of RPBs. However, their related computational cost is very high, as the high packing porosities and complex structures require an extremely fine mesh to capture the minor flow details inside the packing. For these reasons, a limited number of CFD investigations that relied on microscale modelling have been found in literature^13,17,22–29^.

The microscale modelling is crucial for a better understanding of fluid flow behavior within RPBs and for accurately quantifying flow patterns^30^, dry pressure drop^31^, mass transfer rate^31^, efficient operating ranges^32^ and power consumption^31^. Recently, Liu et al.^33^ conducted full-scale simulations using the real geometry of a wire mesh packing in RPB. Their computational grid size consists of 93 million cells in order to obtain detailed hydrodynamic information of single-phase flow inside the wire mesh packing. They managed to study the pressure drop distributions and some local hydrodynamics such as the gas side-end effect zone. However, their investigation was limited to the packing part of RPB and no information was given on the flow characteristics in the inner cavity and the casing. The latter is found to have an important impact on the inner cavity pressure drop^34^ and gas maldistribution factor^16^.

In this study, a microscale CFD model is built to study the flow behavior in the different parts of a RPB. The latter is the one that was the subject of the experimental work by Liu et al.^33^. The gas flow characteristics in the different parts of RPB namely casing, packing and inner cavity are thoroughly analyzed to provide a further understanding of gas misdistribution and free vortex within RPB flow. Moreover, a simplified analytical model is proposed and used to mimic the impact of wire mesh packing on gas flow behavior. This simplified model is used also to verify the validity of viscous and inertial resistance coefficients that are used in macroscale modelling.

## Geometry and Mesh

**Figure 1:**
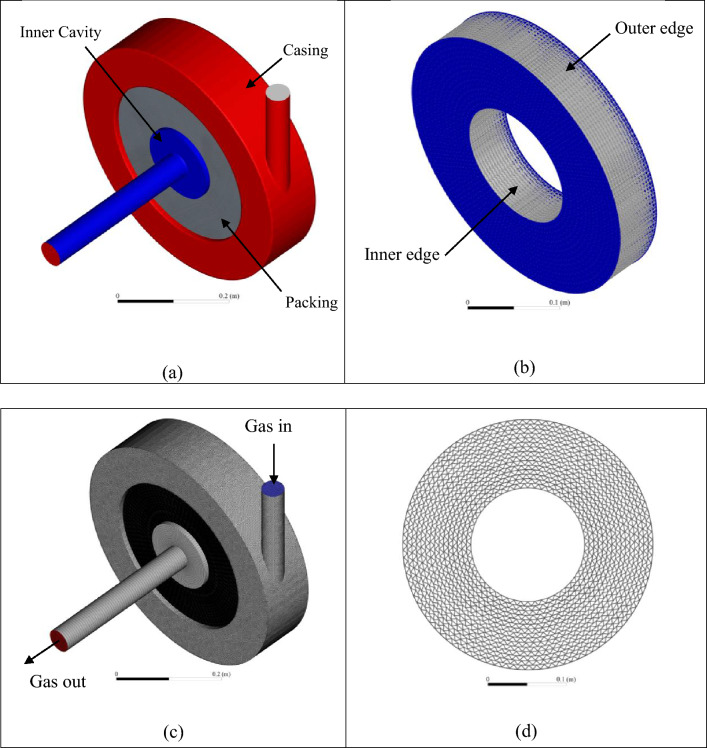
Views for the RPB model of Liu et al.^33^: (**a**) different parts of RPB, including casing, packing, and inner cavity. (**b**) Isometric view of wire mesh packing showing inner and outer edges. (**c**) Mesh views of the RPB’s different parts showing gas inlet and outlet locations. (**d**) Side view of the wire mesh packing showing the wire mesh structure.

The RPB geometric model used for the micro-scale simulations is the one that has been numerically and experimentally studied by Liu et al.^33^. The RPB geometric model is shown in Fig. 1. It comprises a rotor containing a wire mesh packing (shown in Fig. 1b and d, and a stator to represent the outer casing (shown in red color in Fig. 1a). In the eye of the packing (shown in blue color in Fig. 1a), there is an inner cavity region or the so-called free rotation zone, which includes the liquid distributor and the gas outlet.

**Figure 2:**
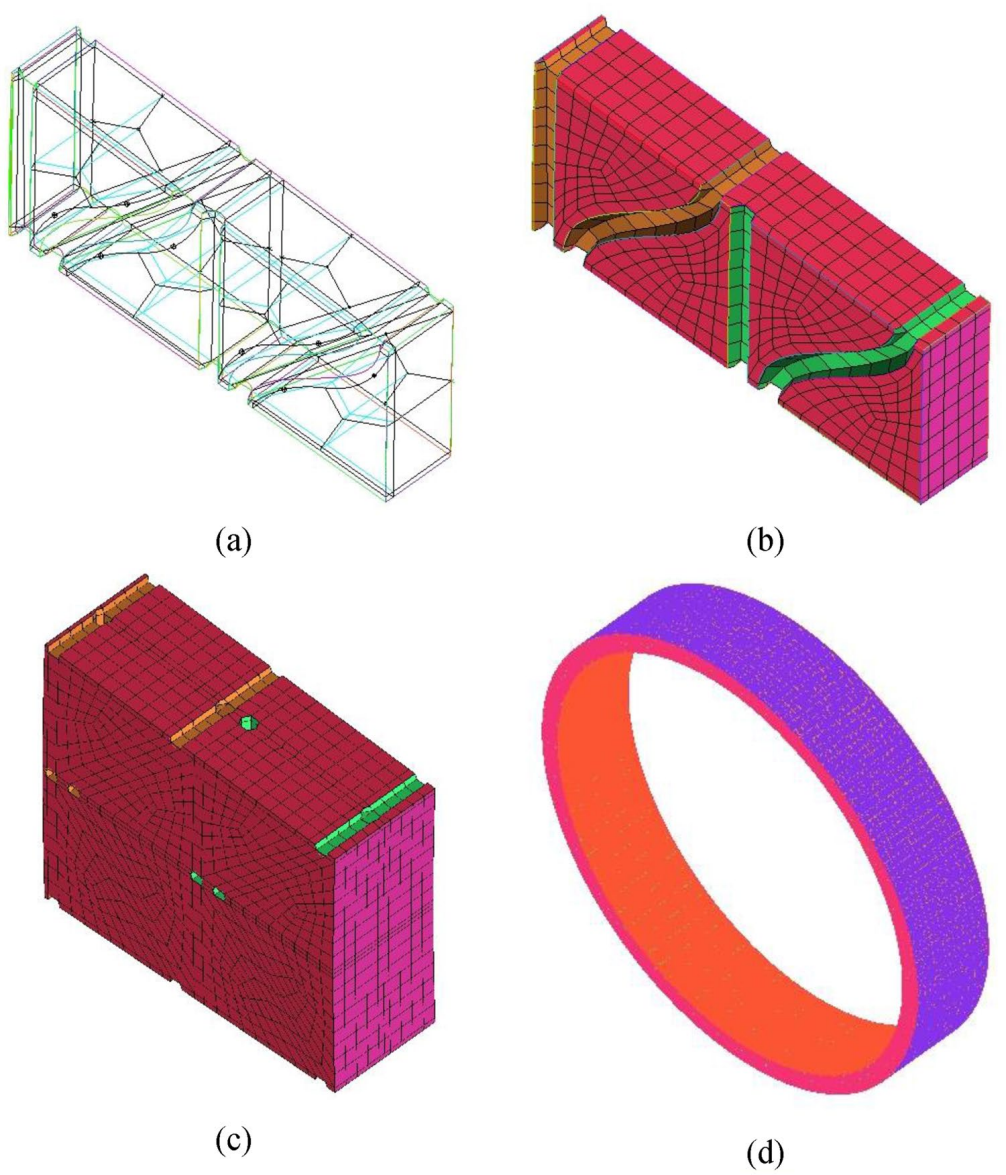
Meshing stages for one array/row of wire mesh packing of Liu et al.^33^: (**a**) the final blocking strategy used to mesh the smallest periodic element. (**b**) Mesh view of the smallest periodic element. (**c**) Mirroring the mesh element in the axial and radial directions. (**d**) Duplicating the mesh element obtained from the previous step around the axial direction to form a closed circular portion.

**Figure 3:**
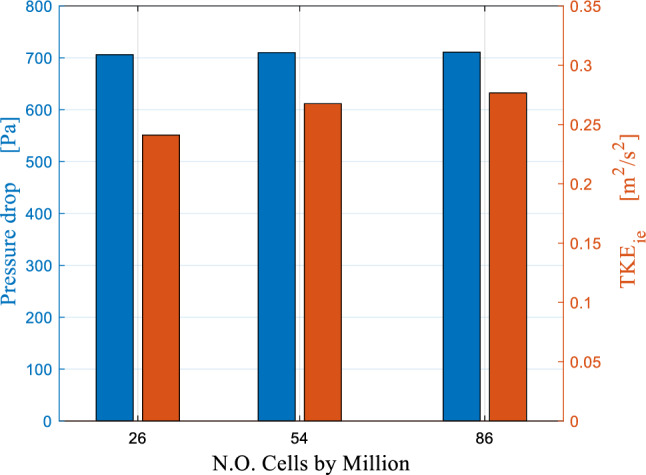
Mesh independence study based on the overall RPB pressure drop as well as TKE near the inner edge under three different grids.

As the wire mesh packing is a large complex geometry with many internal cavities, its meshing process was not a straightforward exercise. Therefore, a repetitive geometry algorithm for mesh processing and setup is proposed. It is summarized herein. First, the packing was discretized into seven concentric arrays/rows. The latter are constructed by duplicating the smallest periodic element in them. ANSYS ICEM is used to construct a structured mesh for only a quarter of each element. Different blocking strategies for a single element quarter have been made prior to reaching the final blocking strategy shown in Fig. 2a. The final blocking strategy could improve the overall mesh quality to reach a minimum orthogonality of 34% with a maximum aspect ratio of about 10. Then, ICEM is used to mirror the mesh of the quarter element (see Fig. 2b) around Y and Z coordinates to obtain a complete periodic element as shown in Fig. 2c. The meshed element was duplicated around the packing axial axes (Z-coordinate), and then they all stitched together to form a closed circular portion. Eventually, this fully circular portion is duplicated in the axial direction. Thus gives a number of identical axial portions where they all stitched together to form a full array as shown in Fig. 2d. The same procedure is repeated for the other elements in each array. Figure 3 shows the results of the grid independence exercise. The latter is conducted using three different resolutions namely, 26, 54, and 86 million cells. It can be seen that the difference in the predicted results of the pressure drop is decreasing as the resolution is increased (0.6% between 26 and 54M resolutions and only 0.1% between 54 and 86M resolutions). For the average radial TKE calculated near the inner edge (r = 0.075 m), the difference in the predicted TKE values is 10% between 26 and 54M resolutions and it drops to 3% between 54 and 88M resolutions. Therefore, 54M resolution is retained as a grid resolution that should balance both accuracy and execution time for the simulations of RPB flow characteristics. It is worth noting that 49M cells are used to mesh the packing region, 4.5M for the outer casing, and 0.12M for the inner cavity region. ANSYS Design Modeler is used to generate unstructured mesh (packing and outer casing) and structured mesh (inner cavity region).

## Mathematical model

The gas flow through an unirrigated packing is considered steady and incompressible. Mass and momentum conservation equations for a single-phase flow are:

1 $$ \frac{\partial \rho }{{\partial t}} + \frac{\partial }{{\partial x_{j} }}\left( {\rho u_{j} { }} \right) = 0 $$

2 $$ \frac{{\partial \overline{{u_{i} }} }}{\partial t} + \overline{{u_{j} }} \frac{{\partial \overline{{u_{i} }} }}{{\partial x_{j} }} = \frac{ - 1}{\rho }\frac{\partial P}{{\partial x_{i} }} + \frac{\partial }{{\partial x_{j} }}\left[ {\nu \left( {\frac{{\partial \overline{u}_{j} }}{{\partial x_{i} }} + \frac{{\partial \overline{u}_{i} }}{{\partial x_{j} }}} \right) - \overline{{u_{i}{\prime} u_{j}{\prime} }} { }} \right] + g_{i} + \frac{{S_{F,i} }}{\rho }{ } $$

The k–ε turbulence model has been widely used in micro-scale CFD simulations of RPB by many previous studies^33,35–39^. This turbulence model has the ability to validate rotating and free shear flow, channel flow, and boundary layer flow with and without pressure gradient^40^. Reynold stress term $\overline{{u_{i}^{\prime } u_{j}^{\prime } }}$ is modelled by the turbulent viscosity concept and hence we can close the ensemble-averaged Navier Stokes equation, (Eq. 2). The turbulent viscosity and length scales are obtained using Boussinesq hypothesis^41^ and based on the solution of two transport equations.

3 $$ \tau^{\prime}_{ij} = - \rho \overline{{u_{i}^{\prime } u_{j}^{\prime } }} = \mu_{t} \left( {\frac{{\partial \overline{u}_{j} }}{{\partial x_{i} }} + \frac{{\partial \overline{u}_{i} }}{{\partial x_{j} }}} \right) $$

The transport equations represent the conservation of turbulence kinetic energy ($k$) and turbulence kinetic energy dissipation rate ($\varepsilon$). Both are described as:

4 $$ \overline{u}_{j} \frac{{\partial \left( {\rho k} \right)}}{{\partial x_{j} }} = \frac{\partial }{{\partial {\text{x}}_{j} }}\left[ {\left( {\mu + \frac{{\mu_{t} }}{{\sigma_{k} }}} \right)\frac{\partial k}{{\partial {\text{x}}_{j} }}} \right] + P_{k} - \rho \varepsilon $$

5 $$ \overline{u}_{j} \frac{{\partial \left( {\rho \varepsilon } \right)}}{{\partial x_{j} }} = \frac{\partial }{{\partial {\text{x}}_{j} }}\left[ {\left( {\mu + \frac{{\mu_{t} }}{{\sigma_{\varepsilon } }}} \right)\frac{\partial \varepsilon }{{\partial {\text{x}}_{j} }}} \right] + C_{1\varepsilon } \frac{\varepsilon }{k}P_{k} - \rho C_{2\varepsilon } \frac{{\varepsilon^{2} }}{k} $$

where $P_{k}$ is rate of turbulence production and is given (Boussinesq hypothesis^41^) as:

6 $$ P_{k} = - \rho \overline{{u_{i}^{\prime } u_{j}^{\prime } }} \frac{{\partial \overline{u}_{i} }}{{\partial x_{j} }} = \mu_{t} \left( {\frac{{\partial \overline{u}_{j} }}{{\partial x_{i} }} + \frac{{\partial \overline{u}_{i} }}{{\partial x_{j} }}} \right)\frac{{\partial \overline{u}_{i} }}{{\partial x_{j} }} $$

where $\sigma_{k}$ = 1 and $\sigma_{\varepsilon }$ = 1.3 for boundary layer flow. $C_{1\varepsilon }$ = 1.44 and $C_{2\varepsilon }$ = 1.92^42^. The eddy viscosity is obtained from the following equation:

7 $$ \mu_{t} = \rho C_{\mu } \frac{{k^{2} }}{\varepsilon } $$

where $C_{\mu }$ equals to 0.09^42^.

At the gas inlet, a 5% turbulent intensity is assumed. A scalable wall function was used for inner cavity and casing walls to ensure Y + requirement for the k– ε turbulence model. A rotating reference frame was adopted herein to model the packing rotation. This helps significantly reduce the computational time if compared to the use of a sliding mesh. As a result, two additional acceleration terms are added to momentum Eq. (2): the Coriolis acceleration: $2\vec{\omega } \times \overrightarrow {{V_{r} }}$ and the centripetal acceleration:$\vec{\omega } \times \vec{\omega } \times \vec{r}$. These two terms are implicitly included in the gravitational term as follows:

8 $$ \vec{g} = 2\vec{\omega } \times \overrightarrow {{V_{r} }} + \vec{\omega } \times \vec{\omega } \times \vec{r}{ } $$

All CFD simulations were conducted using FLUENT 2022—R1 commercial software (https://www.ansys.com/products/fluids/ansys-fluent). A second-order discretization scheme for continuity, momentum, and turbulence transport equations was used based on SIMPLE algorithm for pressure velocity coupling. The solution was initially iterated for 2000 iterations using an inviscid flow condition (initial solution) before running the chosen turbulence model. The solution is considered to be converged when the RMSE of all field variables: velocity, turbulence kinetic energy, turbulence dissipation rate, and pressure achieve their minimum levels of 1e−5. In addition to that, the average pressure was also monitored until it remained almost unchanged at the gas inlet, which finally resulted in a total of 5000 iterations. Calculations were carried out on the Almesbar HPC cluster (https://www.ku.ac.ae/research-offices/research-computing) of Khalifa University using one computing node, where each node has 2 CPUs, and each CPU has Intel® Xeon® Gold 6230-R @ 2.1 GHz with 26 cores and 384 GB RAM.

## Model validation

The validation of the CFD model shows that the predicted pressure drop using the micro-scale model matches well the experimental data reported by Liu et al.^33^. The discrepancies in terms of root mean square error (RMSE) lie within an acceptable range. Saying that, the CFD results show also a small over-prediction of the pressure drop at low rotating speeds and a slight under-prediction at higher rotating speeds.

**Figure 4:**
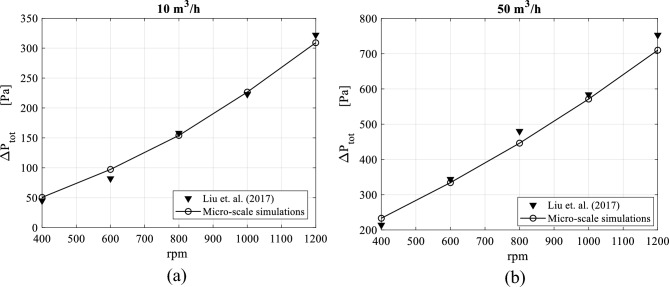
Comparison between CFD and experimental pressure drops versus packing rotating speed for two gas flow rates: (**a**) gas flowrate = 10 m^3^/h. (**b**) Gas flowrate = 50 m^3^/h.

**Figure 5:**
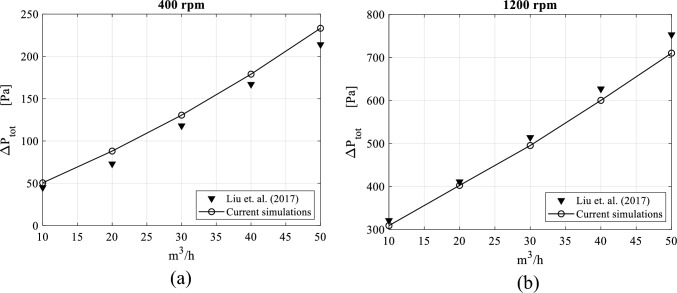
Comparison between CFD and experimental pressure drops against gas flow rate for two rotational speeds: (**a**) rotating speed = 400 rpm. (**b**) Rotating speed = 1200 rpm.

Figure 4 compares the total pressure drop as predicted by the CFD model with the experimental results of Liu et al.^33^ for two different gas flowrates: 10 and 50 m^3^/h. Figure 5 presents a similar comparison but for two rotating speeds: 400 and 1200 rpm.

**Figure 6:**
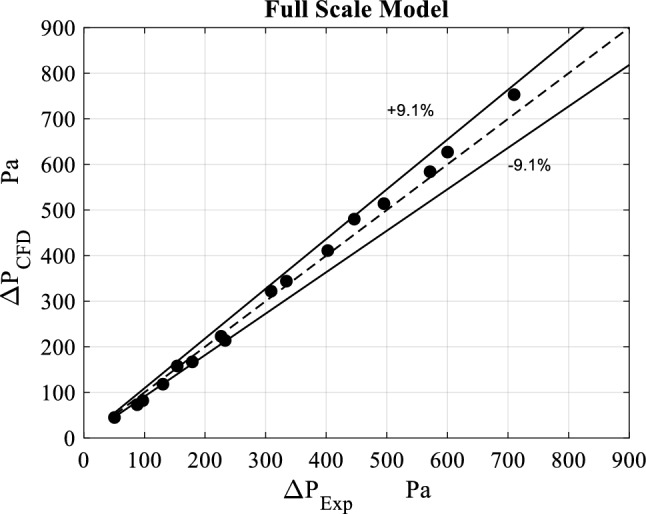
Error analysis of pressure drop values obtained from CFD simulations against experimental results^33^.

Figure 6 provides a comparison between the micro-scale CFD model and the corresponding experimental results for different operating conditions. It can be seen that the relative errors are within 10% except for two cases: the first case is the one for 10 m^3^/h volume flowrate and 400 rpm rotating speed and the second is for 10 m^3^/h volume flowrate and 600 rpm rotating speed as their relative errors reach 20%.

ANSYS CFD-Post was employed to post-process and analyze the CFD model results. Command editor along with MATLAB codes are used to generate infinity numbers of sector planes, circles, and tabular data. The results related to each part of RPB (Casing, wire mesh, and inner cavity) are displayed separately and the flow behavior within them is analyzed. The final data is presented in the supplementary material part in Excel format. The data addresses the different parts of the RPB and the entire operating condition. It also presents Cas and Dat files for one simulation case running at 400 rpm and 30 m^3^/h.

## Casing analysis

**Figure 7:**
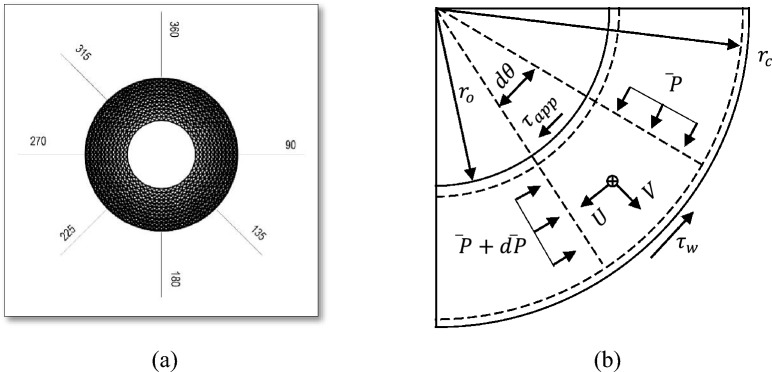
One-dimensional model for the gas flow in the outer cavity: (**a**) XY plane views of the different tangential sections in the outer cavity. (**b**) The control volume used to deduce the one-dimensional model.

Understanding the flow characteristics in the outer cavity of an RPB has received attention nowadays. However, the current investigations are limited to studying gas pressure drop in the outer cavity^33,43^ as well as developing new designs for the housing section to obtain a better gas distribution^15,16^. In the current study, we carried out an extensive investigation to know deeper the flow behavior and related phenomena such as gas maldistribution in the outer cavity (casing). Regarding this, a simplified linear momentum and mass conservation model has been developed for a small angular control volume. The boundaries for such a model are shown in Fig. 7b.

Mass conservation:

9 $$ \frac{{d\overline{U}\left( \theta \right)}}{d\theta } = \frac{{r_{o} }}{{r_{c} - r_{o} }}\overline{V}\left( \theta \right) $$

Momentum conservation:

10 $$ 2\rho \overline{U}\left( \theta \right)\frac{{d\overline{U}\left( \theta \right)}}{d\theta } = \left( {\frac{{r_{o} \tau_{app} - r_{c} \tau_{c} }}{{r_{c} - r_{o} }}} \right) - \frac{{d\overline{P}\left( \theta \right)}}{d\theta } $$

Equation (10) can be further simplified by assuming a constant circumferential pressure profile (uniform pressure) around the packing as deduced from Fig. 12a. Combining Eqs. (9) and (10) results in:

11 $$ 2\rho \overline{U}\left( \theta \right)\overline{V}\left( \theta \right) = \left( {\tau_{app} - \frac{{r_{c} }}{{r_{o} }}\tau_{c} } \right) $$

where:

$\tau_{app}$: is the apparent shear stress induced by the rotating packing on the gas stream inside the casing.

$\tau_{c}$: is the wall shear stress on the casing walls.

$\overline{P}$,$\overline{U}$ and $\overline{V}$ are the average pressure, circulation velocity (average circumferential gas velocity within casing) and radial velocity (average radial velocity at the outer periphery of the packing). Its direction is given in Fig. 7b, and packing radial velocity at outer edge, respectively. They are averaged in the radial and axial directions.

$r_{c}$: Casing inner radius.

$r_{o}$: Packing outer radius.

The gas maldistribution factor within the packing region can be computed^16^ as:

12 $$ rM_{f}^{B} \left( {r, h, \theta } \right) = \frac{1}{{n_{t} }} \mathop \sum \limits_{ }^{{n_{z} }} \mathop \sum \limits_{ }^{{n_{\theta } }} \left( {\frac{{u_{r} \left( {r, z, \theta } \right)}}{{\overline{u}_{r} \left( r \right)}} - 1} \right) $$

where: $\overline{u}_{r} \left( r \right)$ is the average radial velocity at a radial location $r$. $u_{r} \left( {r, z, \theta } \right)$ is the local radial velocity at each collecting point (*r*, *z*, θ). $n_{t}$ is the number of collecting points.

where $$n_{t} = n_{z} \left( {{\text{axial}}} \right) \times n_{\theta } \left( {t{\text{angential}}} \right)$$.

**Figure 8:**
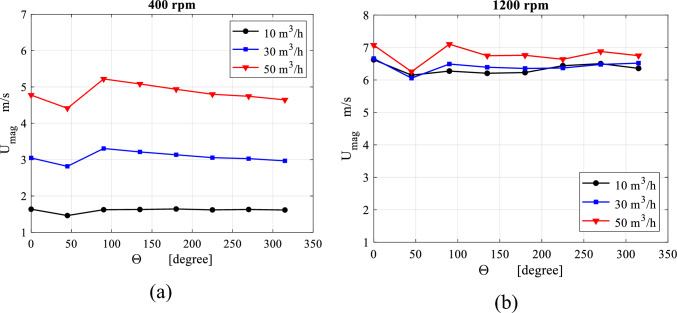
Distribution of the mean gas circulation velocity in the outer cavity, which has derived from the CFD model, over a number of selected tangential sections at different gas flowrates: (**a**) rotating speed = 400 rpm. (**b**) Rotating speed = 1200.

Figure 8 shows the average velocity profiles in the outer cavity region at different angles surrounding the packing periphery (see Fig. 7a). At low rotating speed, the gas circulating velocity in the casing increases by increasing the inlet gas inertia (inlet gas flowrate). Meanwhile, the circulation velocity in the casing increases between 45° and 90° and then it decreases in the remaining parts of the casing. At high rotating speeds, the gas flow in the casing becomes more affected by the packing rotational speed than the inlet gas flow rate. Herein, the circulating gas in the casing accelerates in the section between 45° and 90° and decelerates in the region between 90° and 135° followed by a fluctuating pattern in the remaining section.

**Figure 9:**
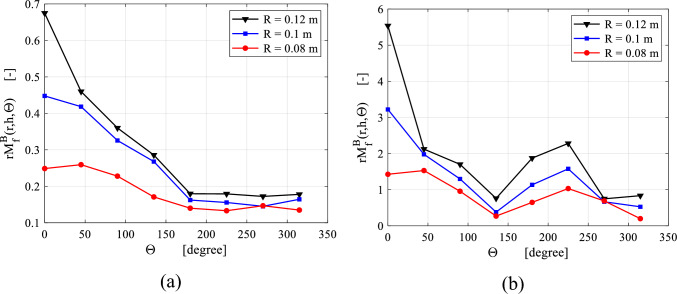
Gas maldistribution factor obtained from Eq. (12) at three mid packing locations for two different cases: (**a**) at rotating speed of 400 rpm and gas flowrate of 30 m^3^/h. (**b**) At rotating speed of 1200 rpm and gas flowrate of 30 m^3^/h.

**Figure 10:**
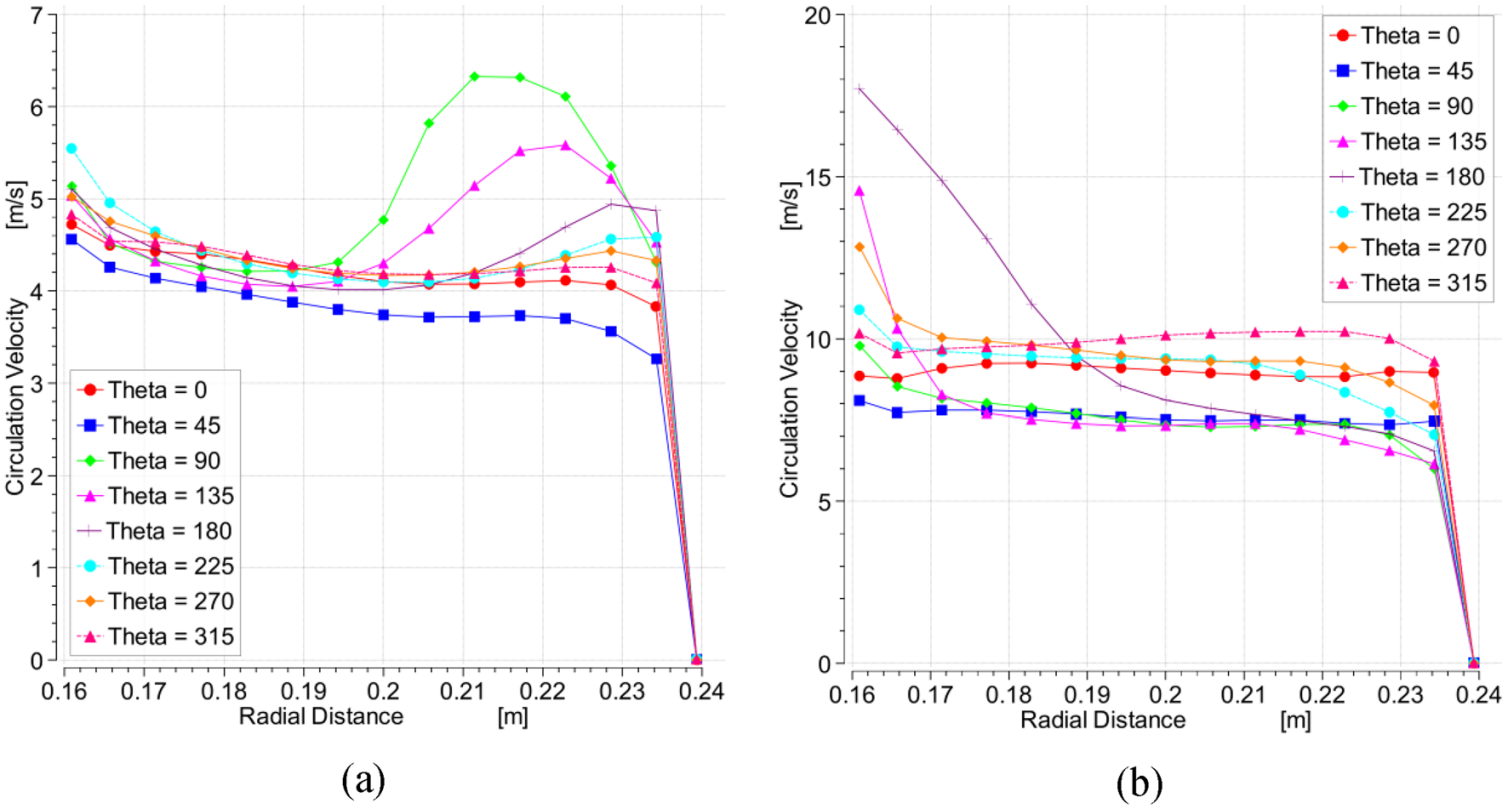
Radial profiles of the gas circulation velocity in the outer cavity at the centerline location and at a number of selected tangential sections for two different cases: (**a**) at rotating speed of 400 rpm and gas flowrate of 30 m^3^/h. (**b**) At rotating speed of 1200 rpm and gas flowrate of 30 m^3^/h.

Figure 9 shows the gas maldistribution factor at different angular locations surrounding the wire mesh packing. The highest profile of gas maldistribution factor is achieved in the vertical position at zero angle (see Fig. 7a). Then, it decreases as gas circulates in a clockwise direction. A reverse flow is expected at the outer periphery of the packing between 45° and 90°, where the gas flow is accelerated. This result can be deduced from Eq. (9) as $\overline{V}$ will be outward when $\frac{{d\overline{U}\left( \theta \right)}}{d\theta }$ is greater than zero. So, this part of the packing (between 45° and 90°) would support the gas maldistribution factor as depicted in Fig. 9a. However, the decelerating flow in the remaining section of the casing would reduce the maldistribution phenomena since $\left( {\frac{{d\overline{U}\left( \theta \right)}}{d\theta } < 0} \right)$ as depicted in Fig. 9a. This is the case at low rotating speeds, while at higher rotating speeds this only applies to the region between 90° and 135°. In the remaining section, the gas maldistribution factor shows a slight increase before decreasing again, as illustrated in Fig. 9b. In order to understand this behavior, the circumferential gas velocity in the outer cavity is investigated along the radial direction as shown in Fig. 10b. At high rotating speed, the circumferential gas velocity in the vicinity of the packing outer edge $(R = 0.16$) approaches the rotor circumferential velocity, while as we move away from the packing, the gas velocity decreases. This means that the momentum of the inlet gas is not sufficient to keep pace with the packing rotating velocity such as in Fig. 10a. This leads to an increase in the velocity gradient of the circulation gas near the packing outer edge as depicted in Fig. 10b. The apparent shear stress, which is proportional to the velocity gradient in vicinity of the rotating packing, increases until it exceeds the critical value ${ }\left( {r_{o} \tau_{app} \ge r_{c} \tau_{c} } \right)$. At this point, an accelerating flow develops again in the outer cavity between 135° and 225°, while the radial gas velocity becomes positive since $\left( {2\rho \overline{U}\left( \theta \right)\overline{V}\left( \theta \right) \ge 0} \right)$ (see Eq. (11)). Consequently, the gas maldistribution factor would increase between 135° and 225° as seen in Fig. 9b.

As discussed above, the accelerating flow, which takes place in the first part of the outer cavity and partially in the second part, is the main responsible of the reverse flow and thus the gas maldistribution in the studied gas inlet design. To remedy this problem, different gas inlet designs have been suggested such as those proposed by Llerena-Chavez and Larachi^16^. Another solution would be to encapsulate the wire mesh packing with stationary coaxial wires. Thus, the gas circulating velocity in the outer cavity is no longer influenced by the apparent shear stress, which arises from the rotating packing.

## Wire mesh analysis

**Figure 11:**
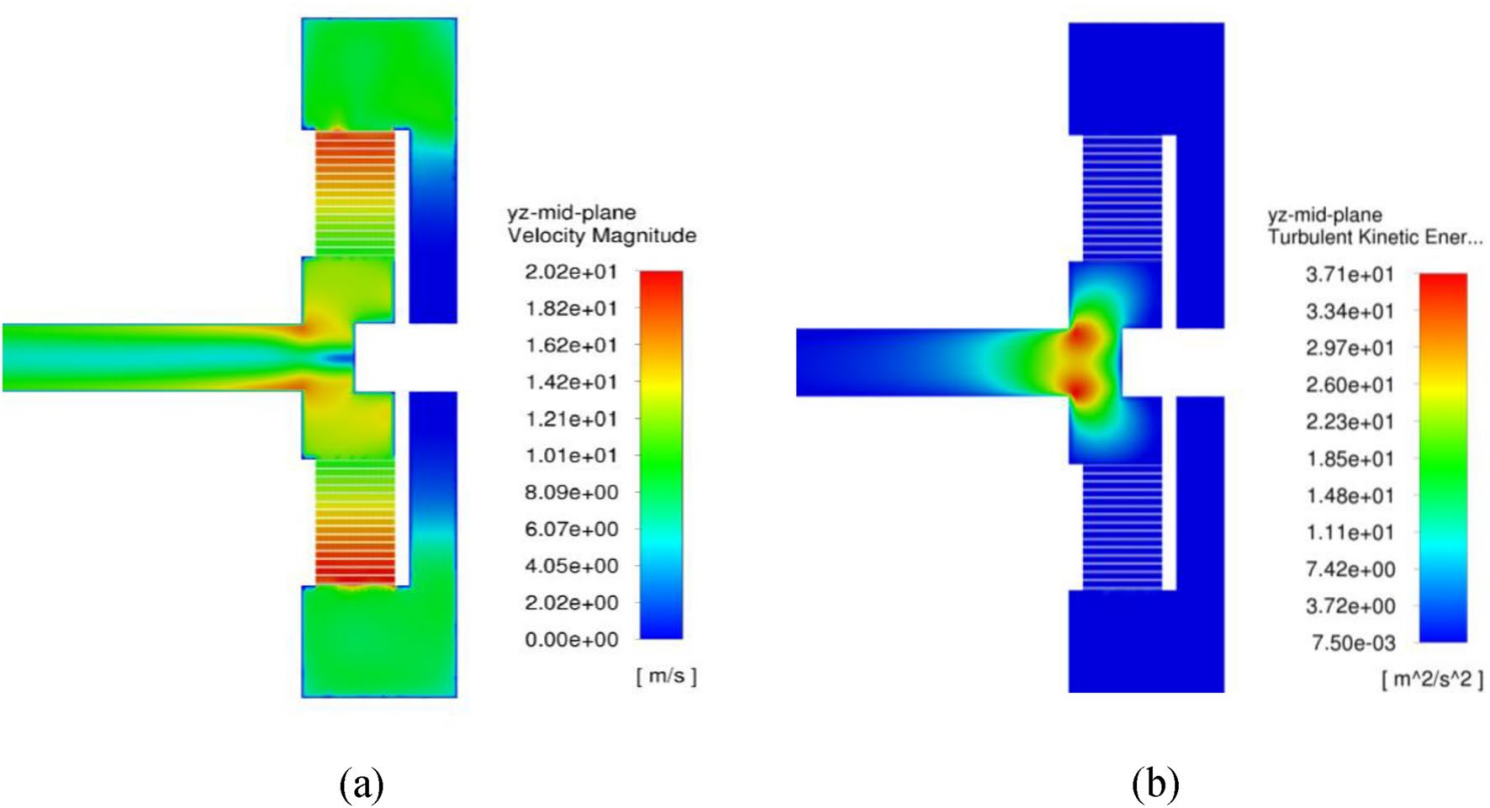
Contour plots at the mid-section of RPB at a rotating speed of 1200 rpm and a gas flowrate of 50 m^3^/h highlighting: (**a**) velocity magnitude at the different RPB’s parts. (**b**) Turbulent kinetic energy at the different RPB’s parts.

**Figure 12:**
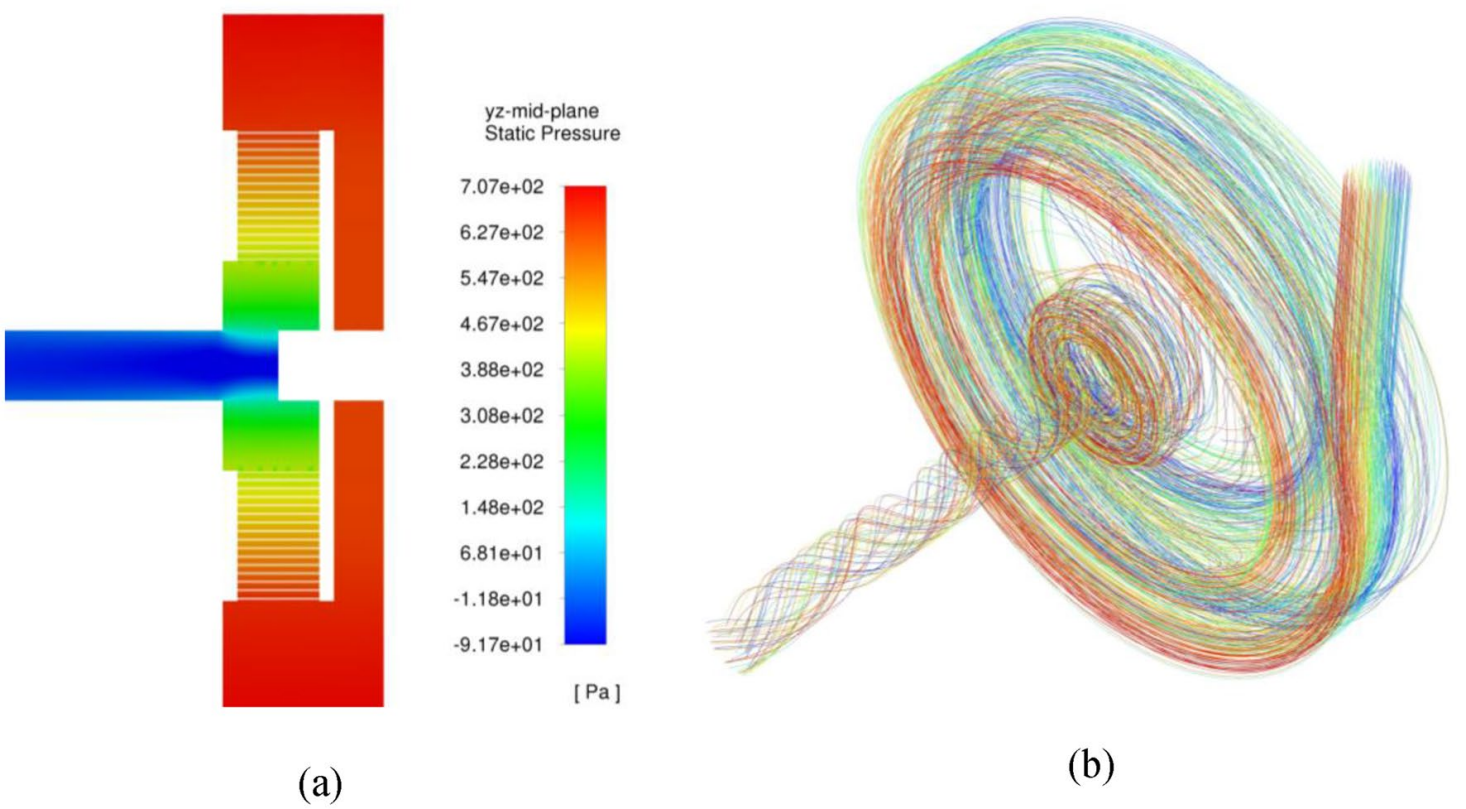
(**a**) Pressure contour plots at the mid-section of RPB, (**b**) streamlines of the gas flow inside the different regions of RPB at a rotating speed of 1200 rpm and a gas flowrate of 50 m^3^/h.

Figure 11 shows velocity magnitude and turbulence kinetic energy contours at 50 m^3^/h and 1200 rpm in the mid-section of the RPB. The maximum velocity magnitude is obtained near the outer edge of the packing, while it decreases as gas flows inward to the inner edge. Two regions of maximum turbulence kinetic energy in the RPB can be noted. The first one is a very narrow area near the packing outer edge and the other one lays in the inner cavity region. The pressure contours at the same operating conditions are shown in Fig. 12a.

The pressure contour plots indicate that the pressure distribution is nearly homogenous in the radial direction inside the packing region as well as in the inner cavity as shown in Fig. 12a. Figure 12b depicts the streamline at different regions inside the RPB: casing, packing and inner cavity. There are many forms of flow encountered in the casing and inner cavity areas, including circulating flow, whirling flow, and free vortex flow.

### Axial profiles

**Figure 13:**
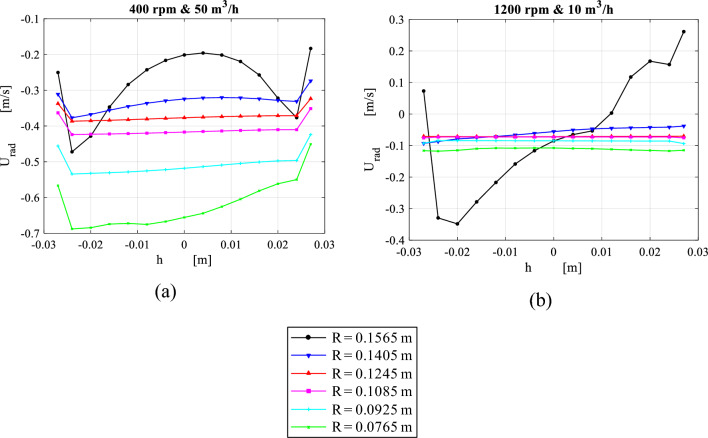
Distribution of circumferential average of radial velocity over axial distance inside the packing at a number of different radial locations for two different cases: (**a**) at rotating speed of 400 rpm and gas flowrate of 50 m^3^/h. (b) At rotating speed of 1200 rpm and gas flowrate of 10 m^3^/h.

Figure 13 depicts the circumferential average of packing radial velocity taken at 15 axial positions under different operating conditions. Initially, the radial velocity is non-uniformly distributed in the axial direction, and some parts of the packing experiences a reverse flow as shown in Fig. 13. As gas flows radially inward, a more uniform radial velocity is achieved. The flow velocity becomes almost fully uniform at a radial distance between 0.1405 and 0.0925 m which is located in the middle part and occupies nearly half of the total packing depth. In other words, flow can be considered as almost two-dimensional flow within this region. As gas advances towards the inner edge, the radial velocity increases. Near the inner edge, the velocity profile becomes non-uniformly distributed whereas the velocity gets higher on one side (inner cavity cap) than the other side. Two different cases are illustrated in Fig. 13: a high gas flow rate at low rotating speed, and a low gas flowrate at high rotating speed. It can be seen that the reverse flow, which takes place at the packing outer edge, is diminished as the ratio of flow velocity to rotating velocity increases.

**Figure 14:**
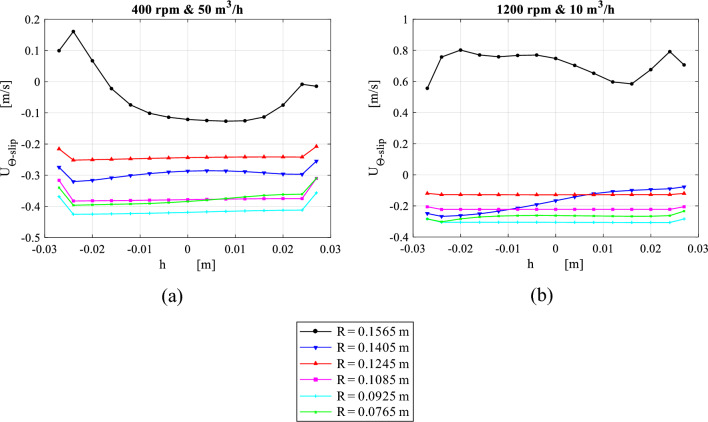
Distribution of circumferential average of absolute tangential velocity over axial distance inside the packing at a number of different radial locations for two different cases: (**a**) at rotating speed of 400 rpm and gas flowrate of 50 m^3^/h. (**b**) At rotating speed of 1200 rpm and gas flowrate of 10 m^3^/h.

Figure 14 displays the circumferential average of the absolute tangential velocity profiles along the axial direction for the same previous cases depicted in Fig. 13. Similar to the radial velocity at the packing entry, the absolute tangential velocity is distributed in a less uniform pattern in the axial direction. However, it becomes uniformly distributed while approaching the packing solid velocity at an earlier radial distance (before $$R = 0.1405)$$. Also, as the gas advances towards the packing inner edge, the slip tangential velocity gains a slight increase due to the increasing Coriolis acceleration. This slight increase in the tangential velocity is clearer for higher gas flowrate. At the inner edge, the absolute tangential velocity becomes slightly higher in the side of inner cavity-cap than the other side (outlet pipe).

**Figure 15:**
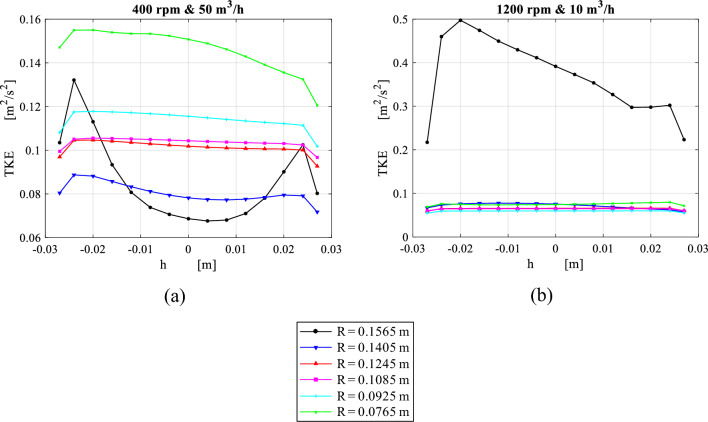
Distribution of circumferential average of TKE over axial distance inside the packing at a number of different radial locations for two different cases: (**a**) at rotating speed of 400 rpm and gas flowrate of 50 m^3^/h. (**b**) At rotating speed of 1200 rpm and gas flowrate of 10 m^3^/h.

Figure 15 shows the circumferential average of turbulent kinetic energy at different radial positions under the same operating conditions. Broadly speaking, the highest TKE levels are observed at both packing wire mesh’s inner and outer peripheries. At high gas flowrate and low rotating speed, TKE values at the inner edge are higher in comparison to its values at the outer edge as shown in Fig. 15a. As the rotating speed increases, TKE level at the inner edge moves to a lower level beneath the one at the outer periphery (see Fig. 15b). It can be seen that TKE levels at the outer edge are tightly related to the slip tangential velocity component (whirling speed), while at the inner edge, they depend mainly on the radial packing velocity. A support of this claim is the high similarity that can be observed between slip tangential velocity and TKE profiles at the outer edge in Figs. 14 and 15, respectively. The same applies to the radial velocity and TKE profiles at the inner edge in Figs. 13 and 15, respectively.

### Radial profiles

**Figure 16:**
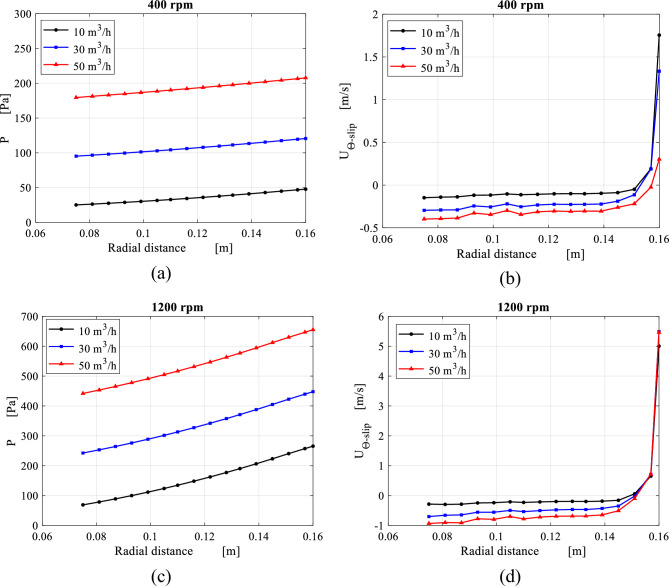
Axial average of pressure as well as slip tangential velocity distributions over radial location inside the packing at different gas flowrates: (**a**) pressure distribution at rotating speed of 400 rpm. (**b**) Slip tangential velocity distributions at rotating speed of 400 rpm. (**c**) Pressure distribution at rotating speed of 1200 rpm. (**d**) Slip tangential velocity distributions at rotating speed of 1200 rpm.

Figure 16 shows the axial-averaged pressure distribution at 16 radial positions. The pressure profile at low rotating speed (400 rpm) has a strict linear trend and most of the pressure drop occurs within the inner cavity. At higher rotating speed (1200) rpm, the pressure profiles show completely different trends due to the increased centrifugal pressure associated with the higher rotating speed. Figure 16b, d depict the axial-averaged slip tangential velocity distribution along the packing radius. It can be seen that the average slip tangential velocity is partially higher than the packing solid velocity. However, the relative tangential velocity of the gas leaving at the inner edge, does not exceed ~ 15% of the corresponding packing solid velocity. This is attributed to the viscous shear forces exerted by the wire mesh in the peripheral direction, which results in retarding the gas tangential velocity.

**Figure 17:**
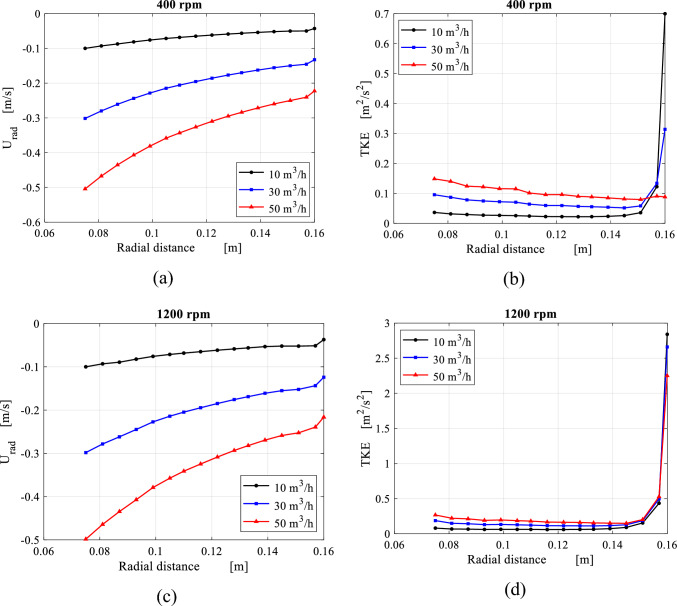
Distributions of axial average of radial velocity and TKE over radial location inside the packing at different gas flowrates: (**a**) radial velocity distribution at rotating speed of 400 rpm. (**b**) TKE distributions at rotating speed of 400 rpm. (**c**) Radial velocity distribution at rotating speed of 1200 rpm. (**d**) TKE distributions at rotating speed of 1200 rpm.

As illustrated in the previous section, the gas flows inside the casing cavity, which surrounds the wire mesh, in the form of a circulating flow. As soon as the outer cavity gas enters the outer periphery of the wire mesh, a sudden increase in the average gas radial velocity component is witnessed near the outer edge as shown in Fig. 17a, c. Similarly, a prompt reduction can be detected in the average turbulence kinetic energy (TKE) levels as shown in Fig. 17b, d. The outer rotating gas enters the packing outer edge at very high turbulence levels $\left( {\frac{{\mathop {\text{u}}\limits^{\prime } }}{{{\overline{\text{U}}}}}} \right. > 50{\text{\% }}$, and consists of mostly large-scale eddies) and once it enters the wire mesh region, the viscous effect surrounding the mesh wires dominates. As a result, TKE dissipates very fast to its minimum level in the vicinity of the outer edge, and this is within less than 10% of the total packing depth. This part of the packing is well known as the gas end effect zone^33^.

Afterwards, a gradual increase in the average TKE is observed while the gas moves towards the inner edge. This indicates that the rate at which the turbulence is generated is higher than the rate at which it dissipates.

**Figure 18:**
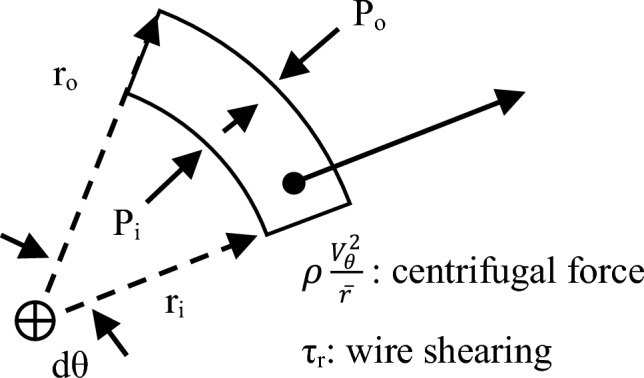
A differential control volume of an annular shape with an inner radius ${\varvec{r}}_{{\varvec{i}}}$ and an outer radius ${\varvec{r}}_{{\varvec{o}}}$ is used to analyze the one-dimensional model for the gas flow in the packing region along the radial direction.

Porous media models are widely used for large-scale RPB simulations to model the wires mesh packing without resolving the details of the flow within it. This is achieved by introducing the so-called packing resistance forces. To mimic the average resistance forces within the wire mesh packing via a micro-scale model, a simplified analytical model is proposed herein. In this model, the radial momentum equation is solved for the radial shear resistance, while the tangential momentum equation is solved for the tangential shear resistance induced by the wires. A control volume of a small annular area (with an inner radius $r_{i}$ and outer radius $r_{o}$) is shown in Fig. 18.

The momentum balance along the radial direction that is based on tangential averaging of flow parameters is given as:

$$ \mathop \sum \limits_{cs} dF_{r} + dF_{B - r} = \dot{m}\left( {\overline{U}_{r,o} - \overline{U}_{r,i} } \right) $$

By substituting for each side:

13 $$ \begin{aligned} & \overline{P}_{i} hr_{i} \cdot d\theta - \overline{P}_{o} hr_{o} \cdot d\theta - \mathop \int \limits_{{r_{i} }}^{{r_{o} }} P \cdot dV + \mathop \int \limits_{{r_{i} }}^{{r_{o} }} \overline{\tau }_{r} \cdot dA_{w} + \rho \mathop \int \limits_{{r_{i} }}^{{r_{o} }} \frac{{\left( {\overline{V}_{\theta }^{ } + \omega r} \right)^{2} }}{r} \cdot dV \\ & \;\; = \rho hr_{av} \overline{U}_{r,av} \left( {\overline{U}_{r,o} - \overline{U}_{r,i} } \right) \cdot d\theta \\ \end{aligned} $$

where:

$$ dV = h \cdot d\theta \cdot rdr $$

$h$ is the packing height.

$$ dA_{w} = a_{p} \cdot dV $$

The momentum change represented by RHS of Eq. (13) is neglected with respect to the centrifugal force (the last term in LHS in Eq. (13)). Thus, Eq. (13) is reduced to:

14 $$ \mathop \int \limits_{{r_{i} }}^{{r_{o} }} \overline{\tau }_{r} a_{p} r \cdot dr = \left( {\overline{P}_{o} r_{o} - \overline{P}_{i} r_{i} - \mathop \int \limits_{{r_{i} }}^{{r_{o} }} P \cdot dr} \right) - \rho \mathop \int \limits_{{r_{i} }}^{{r_{o} }} \left( {\overline{V}_{\theta }^{ } + \omega r} \right)^{2} \cdot dr $$

The wire mesh packing is equally discretized into eight coaxial sectors. Each sector represents an annular control volume. Equation (14) is applied for each sector considering its inner and outer diameters as $r_{i}$ and $r_{o}$, respectively. LHS of Eq. (14) can be approximated by taking the average of $\overline{\tau }_{r}$ over each integration sector $\left( {r_{o} - r_{i} } \right)$. This results in:

15 $$ \overline{\tau }_{r,av} a_{p} = \frac{2}{{r_{o}^{2} - r_{i}^{2} }}\left( {\overline{P}_{o} r_{o} - \overline{P}_{i} r_{i} - \mathop \int \limits_{{r_{i} }}^{{r_{o} }} P \cdot dr - \rho \mathop \int \limits_{{r_{i} }}^{{r_{o} }} \left( {\overline{V}_{\theta }^{ } + \omega r} \right)^{2} \cdot dr } \right) $$

**Figure 19:**
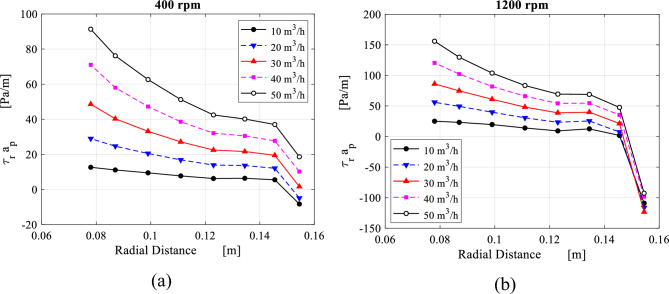
Radial distribution of the packing resistance forces in the radial direction ${\varvec{a}}_{{\varvec{p}}} \overline{\user2{\tau }}_{{{\varvec{r}},{\varvec{av}}}}$ derived from Eq. (15) at the different gas flowrates for two rotating speeds: (**a**) rotating speed of 400 rpm and (**b**) rotating speed of 1200 rpm.

The term $\overline{\tau }_{r} a_{p}$ represents the resistance force per unit of volume induced by the wire mesh packing along the radial direction. Then, the distribution of $\overline{\tau }_{r,av} a_{p}$, which is obtained from applying Eq. (15) at each sector is plotted against the radial direction as shown in Fig. 19.

**Figure 20:**
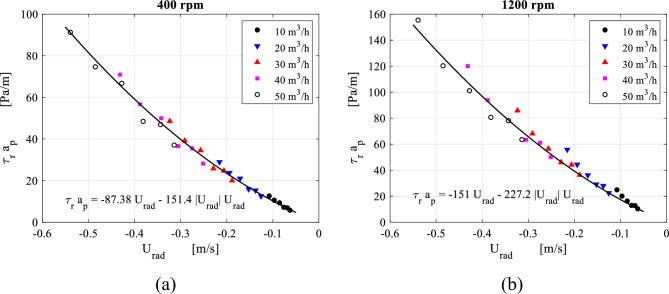
Distribution of the packing resistance forces in the radial direction ${\varvec{a}}_{{\varvec{p}}} \overline{\user2{\tau }}_{{{\varvec{r}},{\varvec{av}}}}$ plotted against radial velocity at the different gas flowrates for two rotating speeds: (**a**) rotating speed = 400 rpm. (**b**) Rotating speed = 1200 rpm.

Both the packing radial resistance per unit of volume $a_{p} \overline{\tau }_{r,av}$ (obtained from Eq. (15)) and the radial flow velocity can be linearly correlated within the packing region as depicted in Fig. 20. This helps verify the validity of Darcy-Forchheimer formula that has been widely used in conventional packed beds. Results displayed in Fig. 20 emphasize the validity of the proposed porous media model in RPBs’ packing for the radial direction in a similar way to conventional packed beds. However, flows in the vicinity of the packing outer edge cannot be governed by the same formula. It seems that is affected by abrupt inlet losses, that are proportional to the packing rotating speed.

**Figure 21:**
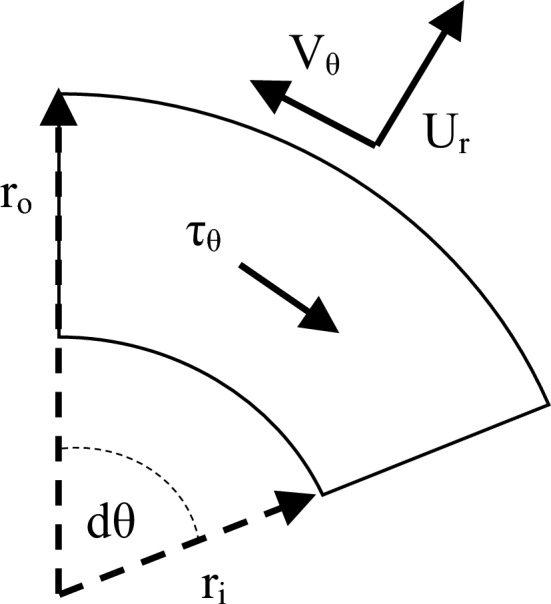
A differential control volume of an annular shape with an inner radius ${\varvec{r}}_{{\varvec{i}}}$ and an outer radius ${\varvec{r}}_{{\varvec{o}}}$ is used to analyze the one-dimensional model for the gas flow in the packing region along the tangential direction.

To solve the tangential shear resistance, we assume a small annular area with infinitesimal angle ($d\theta$) and with an inner radius $r_{i}$ and outer radius $r_{o}$ as shown in Fig. 21. The momentum balance along the tangential direction that is based on tangential averaging of flow parameters is given as:

16 $$ \mathop \sum \limits_{cs} dF_{\theta } + dF_{B - \theta } = \dot{m}\left( {\overline{V}_{\theta ,o} - \overline{V}_{\theta ,i} } \right) $$

By substituting for each term:

17 $$ \mathop \int \limits_{{r_{i} }}^{{r_{o} }} \overline{\tau }_{\theta } \cdot dA_{w} - \rho \mathop \int \limits_{{r_{i} }}^{{r_{o} }} \frac{{\overline{U}_{r} \left( {\overline{V}_{\theta }^{ } + \omega r} \right)^{ } }}{r} \cdot dV = \rho \overline{U}_{r,av} \left( {\overline{V}_{\theta ,o} - \overline{V}_{\theta ,i} } \right)hr_{av} \cdot d\theta $$

where:

$$ dV = h \cdot d\theta \cdot rdr $$

After substituting and rearrangement, Eq. (17) can be rewritten as:

18 $$ \mathop \int \limits_{{r_{i} }}^{{r_{o} }} \overline{\tau }_{\theta } a_{p} \cdot r \cdot dr = \rho \mathop \int \limits_{{r_{i} }}^{{r_{o} }} \overline{U}_{r} \left( {\overline{V}_{\theta }^{ } + \omega r} \right) \cdot dr + \rho \overline{U}_{r,av} \left( {\overline{V}_{\theta ,o} - \overline{V}_{\theta ,i} } \right)r_{av} $$

Equation (18) is applied in each integration sector considering its inner and outer diameters as $r_{i}$ and $r_{o}$, respectively. Then, the above integration can be simplified by averaging $\overline{\tau }_{\theta }$ over the integration interval. Thus, Eq. (18) can be rewritten as:

19 $$ \overline{\tau }_{\theta ,av} a_{p} = \frac{2}{{r_{o}^{2} - r_{i}^{2} }}\left( {\rho \mathop \int \limits_{{r_{i} }}^{{r_{o} }} \overline{U}_{r} \left( {\overline{V}_{\theta }^{ } + \omega r} \right) \cdot dr + \rho \overline{U}_{r,av} \left( {\overline{V}_{\theta ,o} - \overline{V}_{\theta ,i} } \right)r_{av} } \right) $$

**Figure 22:**
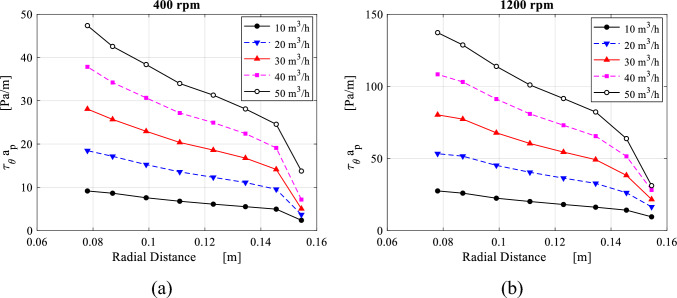
Radial distribution of the packing resistance forces in the tangential direction ${\varvec{a}}_{{\varvec{p}}} \overline{\user2{\tau }}_{{{\varvec{\theta}},{\varvec{av}}}}$ derived from Eq. (19) at the different gas flowrates for two rotating speeds: (**a**) rotating speed of 400 rpm and (**b**) rotating speed of 1200 rpm.

The term $\overline{\tau }_{\theta } a_{p}$ represents the resistance force per unit of volume induced by the wire mesh packing along the tangential direction. Then, the distribution of $\overline{\tau }_{\theta ,av} a_{p}$, which is obtained from applying Eq. (19) at each sector is plotted against the radial direction as shown in Fig. 22.

**Figure 23:**
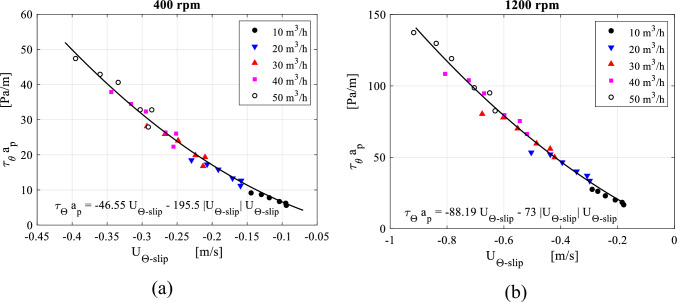
Distribution of the packing resistance forces in the tangential direction ${\varvec{a}}_{{\varvec{p}}} \overline{\user2{\tau }}_{{{\varvec{\theta}},{\varvec{av}}}}$ plotted against slip tangential velocity at the different gas flowrates for two rotating speeds: (**a**) rotating speed = 400 rpm. (**b**) Rotating speed = 1200 rpm.

Similarly, the tangential resistance per unit of volume $a_{p} \overline{\tau }_{\theta ,av}$ caused by the wire mesh packing in the lateral direction of the gas flow can be related to the slip tangential velocity component within the packing region in the form Darcy-Forchheimer equation. Based on data fitting for the results displayed in Fig. 23, we can verify the validity of Darcy-Forchheimer formula in the lateral direction within RPB. The point near the packing outer edge where a sudden increase in the tangential resistance force is witnessed, is excluded.

In a previous work by the Authors^44,45^, a recalibration algorithm for the porous media model coefficients based on Rao et al.^31^ correlation for a given pressure drop between the packing edges and under stationary packing conditions, is derived. In a similar manner, the new fit constants given in Figs. 20 and 23 are used to recalibrate the porous media model.

**Figure 24:**
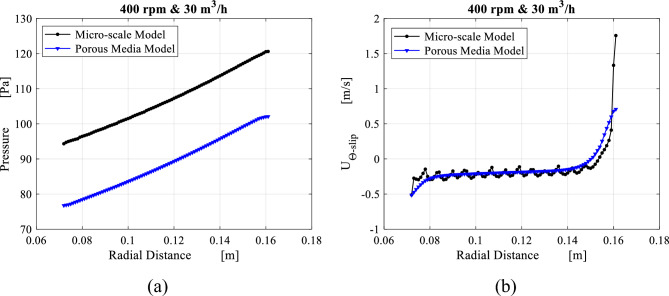
Comparison between results obtained from the microscale model and the porous media model with the new fit coefficients in the packing region in terms of: (**a**) radial distribution of average pressure at 400 rpm and 30 m^3^/h. (a) Radial distribution of average slip tangential velocity at 400 rpm and 30 m^3^/h.

**Figure 25:**
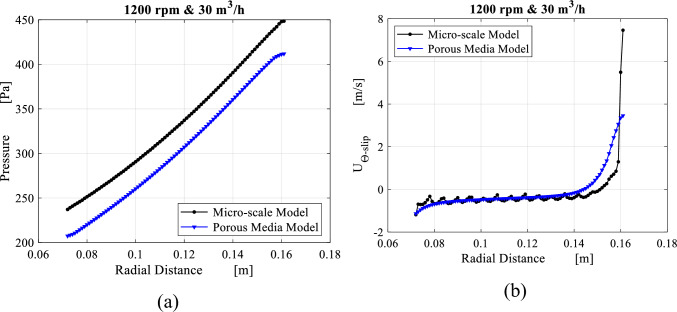
The results of the microscale model against the porous media model with the new fit coefficients including: (**a**) radial distribution of average pressure at 1200 rpm and 30 m^3^/h. (**b**) Radial distribution of average slip tangential velocity at 1200 rpm and 30 m^3^/h.

The porous media model is improved in order to provide results comparable to the ones of the microscale model. The average radial profiles obtained from both models are compared in Figs. 24 and 25 for radial distributions of the pressure and the slip tangential velocity within the packing region for two different operating conditions. Identical trends along both pressure profiles are noted despite the pressure difference at inner edge due to the pressure drop at the inner cavity obtained from both models. Also, slip tangential velocities obtained from both models agree very well in the region between $R = 0.15$ and $R = 0.08$. Although the high level of similarity within the packing, the porous media model is not able to mimic the actual flow conditions as the gas approaches the inner edge. These conditions are the same conditions of the inner cavity entry. As a result, a 10% deviation is observed between the two models in terms of inner cavity pressure drop.

## Inner cavity analysis

The gas leaves the packing inner edge at a circumferential velocity that approaches the packing solid velocity (see Fig. 16b and d). Meanwhile, the gas radial velocity at the inner edge is comparably smaller than the absolute tangential velocity as shown in Fig. 17a and b. Thus, it can be inferred that the gas leaves the inner packing edge almost tangentially. Previous studies have shown that the tangential flow causes a strong free vortex flow inside the inner cavity zone^31,33,34^. However, there are other types of flows in the inner cavity region that were not covered by previous research, as well as how the flow switches from one type to another.

The following simplified momentum equations, which were developed by Zheng et al.^46^ for gas flow in the inner cavity zone of RPB, can help in this regard:

20 $$ \rho u_{r} \frac{{du_{r} }}{dr} - \rho \frac{{V_{\theta }^{2} }}{r} = - \frac{dP}{{dr}} $$

21 $$ \rho u_{r} \frac{{dV_{\theta } }}{dr} + \rho \frac{{u_{r} V_{\theta } }}{r} = - \frac{d\tau }{{dr}} $$

where:

$u_{r}$: is radial velocity component.

$V_{\theta }$: is tangential velocity component.

$\frac{dP}{{dr}}$: is pressure gradient.

$\frac{d\tau }{{dr}}$: is shear stress gradient.

**Figure 26:**
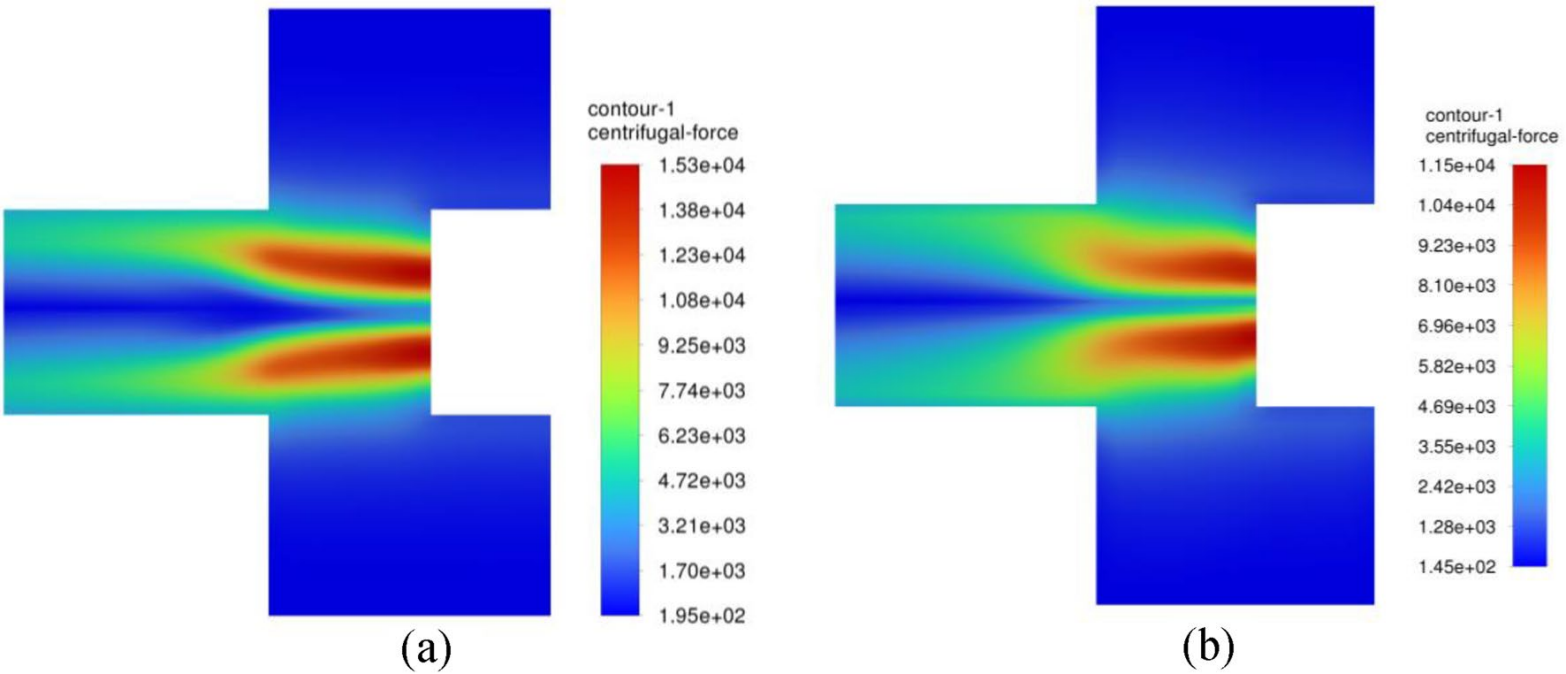
Contour plots of centrifugal acceleration $\left( {{\varvec{\rho}}\frac{{{\varvec{V}}_{{\varvec{\theta}}}^{2} }}{{\varvec{r}}}} \right)$ within the inner cavity region at a packing rotating speed of 400 rpm and gas flowrate of 50 m^3^/h obtained from two different CFD simulations: (**a**) Inviscid flow assumption; (**b**) turbulent flow.

**Figure 27:**
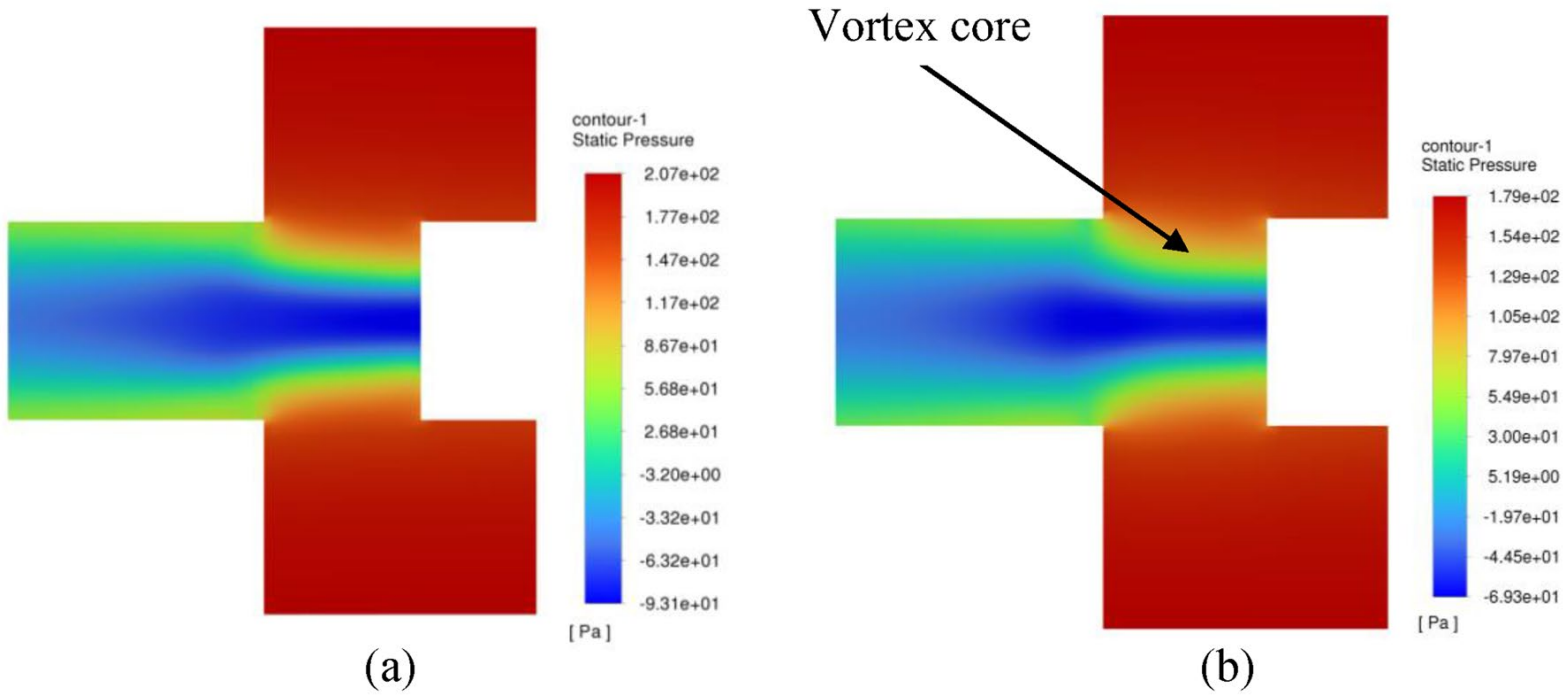
Pressure contour plots within the inner cavity region at a packing rotating speed of 400 rpm and gas flowrate of 50 m^3^/h obtained from two different CFD simulations: (**a**) inviscid flow assumption; (**b**) turbulent flow.

Figure 26 displays the contour plots of the centrifugal acceleration field throughout the inner cavity region. It is seen that the centrifugal gravity increases gradually as gas moves deeper inward. As the gas moves nearer to the entry of the outlet pipe, we can observe two spots of a very strong centrifugal field. Afterwards, they are destroyed as soon as gas flows downstream into the outlet pipe. On the other hand, Fig. 27 presents the corresponding contour plots of static pressure in the inner cavity. The static pressure is, firstly, decreased as the gas moves radially inward into the entry of the outlet pipe. Then, it starts to increase again once the gas passes into the downstream pipe. In the following, the above findings are addressed in light of the previous momentum equations. As the gas moves radially inward through the inner cavity, its radial velocity increases inward $\left( {u_{r} < 0} \right)$. This, in turn, increases the Coriolis acceleration $\left( {\rho \frac{{u_{r} V_{\theta } }}{r}} \right)$, and thereby the gas tangential velocity increases $\left( {\frac{{dV_{\theta } }}{dr} > 0} \right)$ as gas moves radially inward (see Eq. (21)). As a result, a very strong centrifugal acceleration field $\left( {\rho \frac{{V_{\theta }^{2} }}{r}} \right)$ is developed near the entry of the outlet pipe, as shown in Fig. 26b. Meanwhile, the pressure drop increases $\left( {\frac{dP}{{dr}} > 0} \right)$ significantly while the gas is approaching the vortex core as depicted in Fig. 27b (see Eq. (20)).

**Figure 28:**
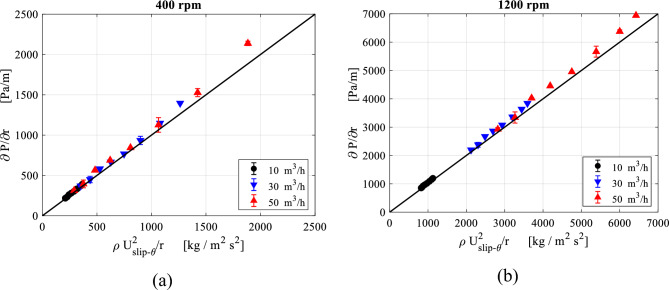
Pressure gradient $\left( {\frac{{{\varvec{dP}}}}{{{\varvec{dr}}}}} \right)$ plotted against centrifugal acceleration $\left( {{\varvec{\rho}}\frac{{{\varvec{V}}_{{\varvec{\theta}}}^{2} }}{{\varvec{r}}}} \right)$ in the inner cavity region at different gas flowrtaes for two rotating speeds: (**a**) rotating speed = 400 rpm; (**b**) rotating speed = 1200 rpm.

The pressure gradient can be correlated with the centrifugal acceleration in this region before reaching the outlet pipe entry, as illustrated in Fig. 28. It is worth mentioning that the pressure gradient values shown in Fig. 28 were obtained by the finite difference approximation method. However, this will result in uncertainties in the estimated derivatives. To estimate these uncertainties in the pressure gradient, an approximation method utilizing Richardson Extrapolation^47^ would be applied. The idea is to find the approximation of the pressure gradient using two grids: a fine grid and a coarse grid. Then, combining the two different grid approximations yields an estimate of the error^48^ (an illustrative example is given in the appendix). Accordingly, the flow in the inner cavity region is considered to be predominantly free vortex flow obsessively between the packing inner edge and the outlet pipe entry. Therefore, the role of viscosity can be considered insignificant in this region. Afterwards, as the gas moves into the vortex core, the flow switches from a free vortex flow into another type of flow so-called swirling flow, and because of this transition an increase in the axial velocity component takes place at the expense of the radial velocity (to achieve the mass conservation). This results in destroying the Coriolis acceleration in this new region, which was the main reason for the free vortex in the former region. To show the importance of the viscosity in this region, we compared two CFD simulations: with and without viscosity effect.

For inviscid flow simulation, which represents the case of no viscous effect (no need for new simulations herein as it was already generated to be used as an initial solution for the turbulent flow simulations), the gas tangential velocity will remain almost unchanged (see Eq. (21)—as there is no longer radial velocity component existed in this region, $\frac{{dV_{\theta } }}{dr} = 0$), and this will keep the pressure reducing at finite rate $\left( {\frac{dP}{{dr}} = {\text{Constant}}} \right)$.

For turbulent flow simulations (with viscous effect), and because of the abrupt change in the main flow direction, an increase in the turbulent kinetic energy is witnessed in this region as indicated in Fig. 31. Since the tangential momentum is conserved (see Eq. (21)), the increase in the turbulent kinetic energy or shear stress $\left( {\frac{d\tau }{{dr}} > 0} \right)$ will result in decreasing the tangential velocity component $\left( {\frac{{dV_{\theta } }}{dr} < 0} \right)$. As a result, the rate at which the pressure is reducing will decrease $\left( {\frac{{d^{2} P}}{{dr^{2} }} < 0} \right)$. Hence, a lower centrifugal force (lower tangential velocity) is obtained in the vortex core of the turbulent flow simulations compared to the inviscid flow simulations as shown in Fig. 26. Moreover, the pressure gradient reduces to zero and becomes favorable pressure gradient $\left( {\frac{dP}{{dr}} < 0} \right)$ more rapidly in the turbulent flow simulations and thereby reaching faster the state of fully established flow as shown in Fig. 27b. Eventually, this reflects the significant role of turbulent viscosity in reducing the pressure drop in the vortex core.

**Figure 29:**
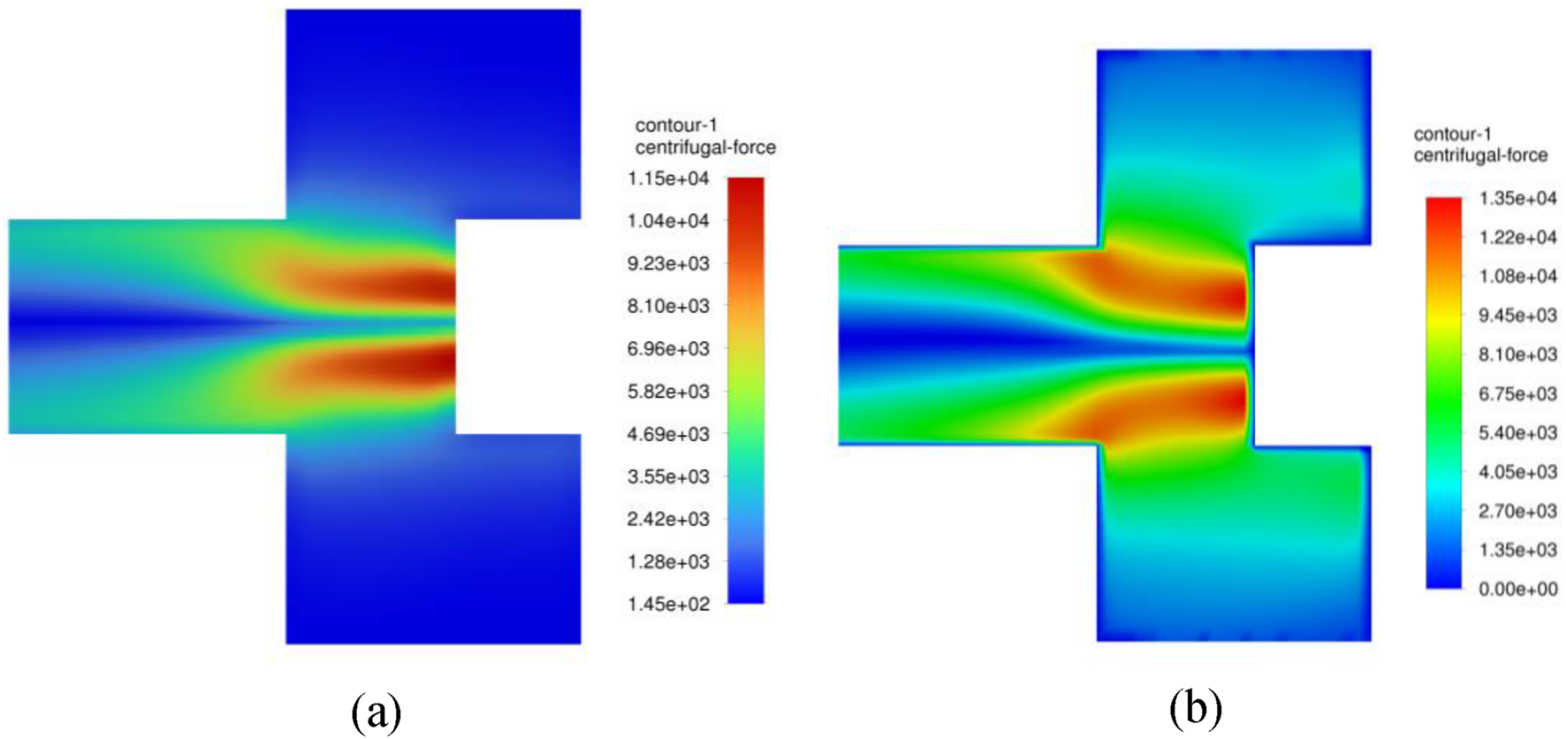
Contour plots of centrifugal acceleration $\left( {{\varvec{\rho}}\frac{{{\varvec{V}}_{{\varvec{\theta}}}^{2} }}{{\varvec{r}}}} \right)$ within the inner cavity region at two different operating conditions: (**a**) rotating speed of 400 rpm and gas flowrate of 50 m^3^/h, (**b**) rotating speed of 1200 rpm and gas flowrate of 50 m^3^/h.

Figure 29 depicts the effect of rotating speed on centrifugal acceleration profiles for the same gas flowrate (50 m^3^/h) in the free vortex region. Although the packing rotating speed is increased three-fold, the maximum centrifugal acceleration is increased only by 17%. This reflects the small effect of the packing rotating speed has on centrifugal acceleration and hence on pressure gradient in this region.

**Figure 30:**
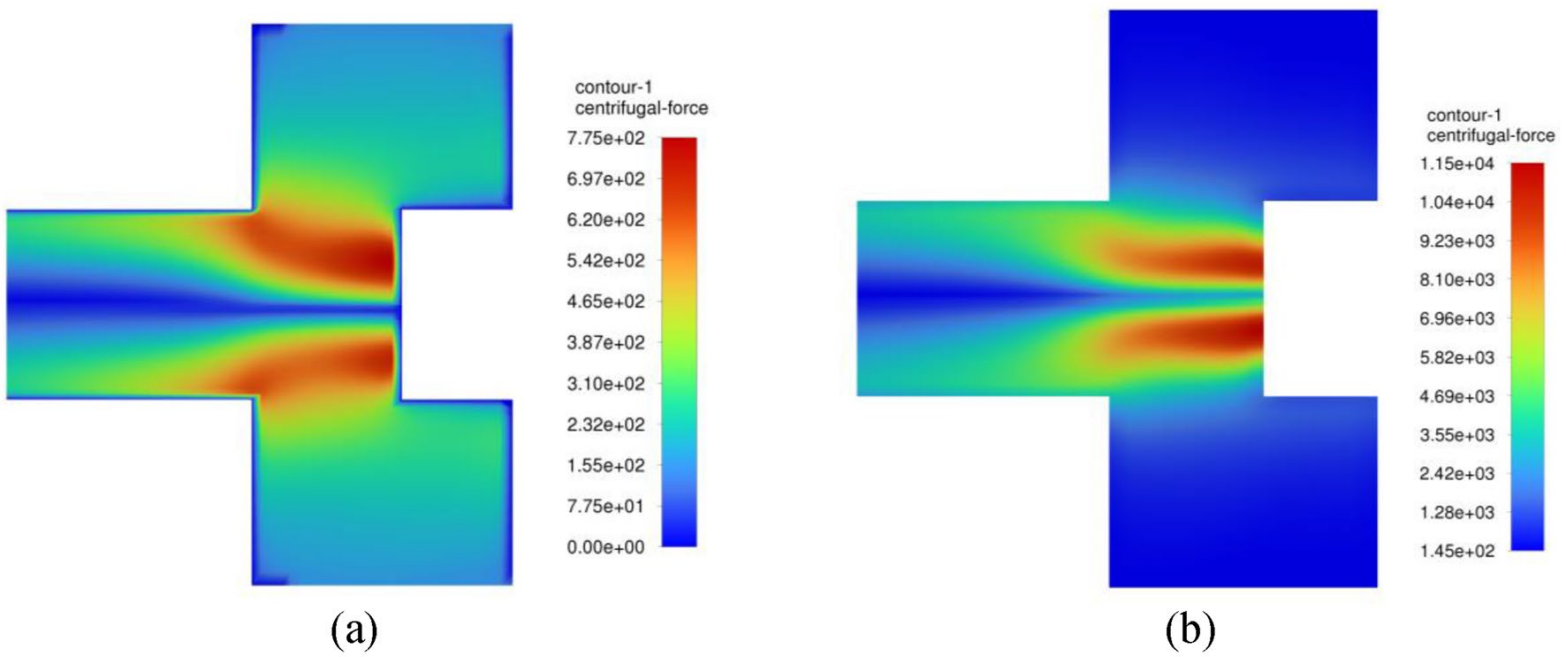
Contour plots of centrifugal acceleration $\left( {{\varvec{\rho}}\frac{{{\varvec{V}}_{{\varvec{\theta}}}^{2} }}{{\varvec{r}}}} \right)$ within the inner cavity region at two different operating conditions: (**a**) rotating speed of 400 rpm and gas flowrate of 10 m^3^/h, (**b**) rotating speed of 400 rpm and gas flowrate of 50 m^3^/h.

Figure 30 depicts the effect of gas flowrate on centrifugal acceleration profiles at the same rotating speed (400 rpm) in the free vortex region. As a result of this increase in the gas flowrate (radial velocity), the maximum centrifugal acceleration is increased by 13.8 times. Recalling that the radial velocity is the main responsible of Coriolis force and thereby free vortex flow. Hence, its impact on centrifugal acceleration and pressure gradient in the free vortex region becomes more significant with increasing the gas flowrate.

**Figure 31:**
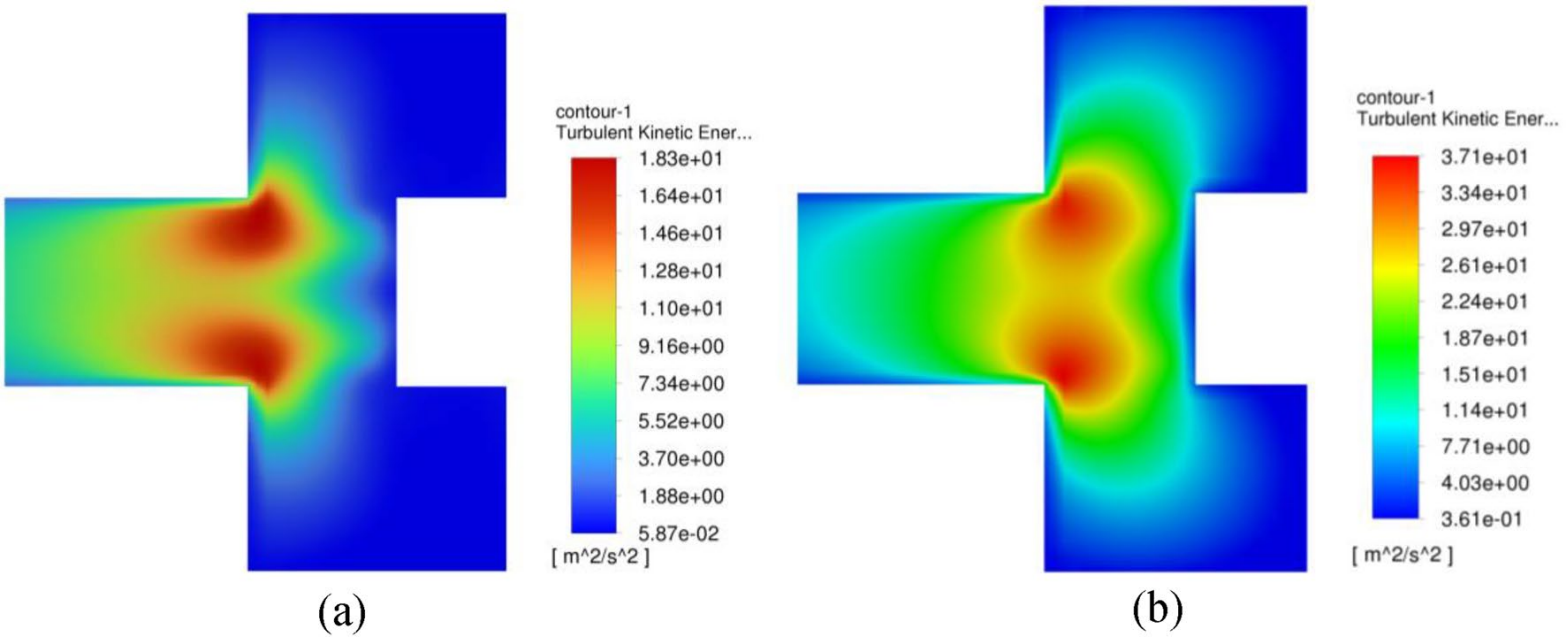
Contour plots of TKE within the inner cavity region at two different operating conditions: (**a**) rotating speed of 400 rpm and gas flowrate of 50 m^3^/h, (**b**) rotating speed of 1200 rpm and gas flowrate of 50 m^3^/h.

**Figure 32:**
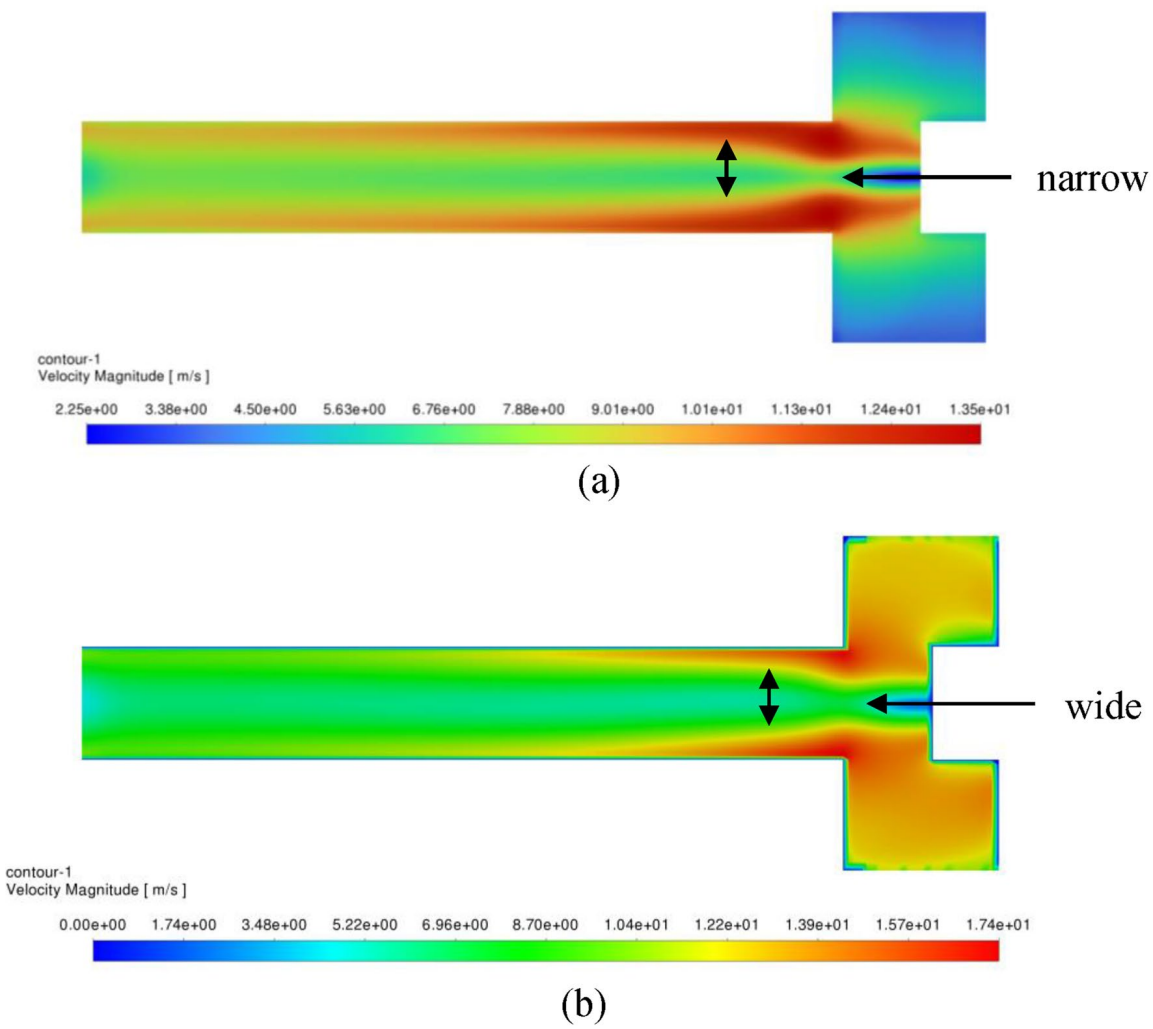
Contour plots of velocity magnitude in the inner cavity region displaying the vortex core size at two different operating conditions: (**a**) rotating speed of 400 rpm and gas flowrate of 50 m^3^/h; (**b**) rotating speed of 1200 rpm and gas flowrate of 50 m^3^/h.

It is worth mentioning that the turbulent kinetic energy, on the other side, controls the tangential velocity component once gas enters the vortex core. Before that, it did not have a significant impact on the free vortex flow. The higher turbulent energy is, the higher shear stress is and hence a rapid retarding of gas tangential velocity in the vortex core (see Eq. (21)). Figure 31 addresses the effect of rotating speed on turbulent kinetic energy for the gas flowrate of 50 m^3^/h in the vortex core. A higher turbulent kinetic energy can be observed near the vortex core as the packing rotating speed is increased. Eventually, the higher turbulent kinetic energy leads to a faster fully established flow in the outlet pipe as it is depicted in Fig. 32.

## Conclusions

RPB technology has been playing an important role in intensifying different chemical and physical processes thanks to the potential of significantly enhancing mass transfer that is induced by the HiGee field. In this study, a microscale CFD model is built and used to investigate the flow behavior within the different parts of a full scale rotating packed bed (RPB). The results on the dry pressure drop agree well with the experimental data available for the set up simulated RPB. The results obtained demonstrate the strong relation between the reverse flow at the outer periphery of the packing and the gas maldistribution factor. The accelerating flow in the inner cavity is the main responsible of the reverse flow and thereby the maldistribution factor. Accelerating flow takes place during the gas feed into the RPB casing and as the rotation of the packing dominates the circulating flow in the casing, a further increase in the gas maldistribution factor is witnessed. It is also found that TKE levels at the packing outer edge are strongly linked to the slip tangential velocity component, while at its inner edge, they depend mainly on the radial packing velocity. The so-called gas end effect zone is detected by observing the TKE profiles near the packing outer edge. The latter accounts for less than 10% of the total packing depth. The validity of the widely used porous media model in RPBs’ packing for both radial and tangential directions was confirmed by the obtained results except for the flow near the packing inner and outer edges. This is attributed to the abrupt change in the local flow conditions at inlet and outlet of the packing, which cannot be well captured by the porous media model. In the inner cavity region, gas flow exhibits two distinctive behaviors. The first one is the free vortex flow. Before reaching the vortex core, gas flow is considered as free vortex dominant flow with a minor effect of the viscosity on the gas flow. Once the gas becomes closer to the vortex core, the turbulence kinetic energy production rate increases and the gas switches from free vortex flow to swirling flow which is the second type of gas behavior. As a result of this transition, the increases in shear stress accelerate the decrease in the gas tangential velocity in the vortex core. This eventually helps speed up the favorable pressure gradient and hence the flow establishment beyond the vortex core. For future investigations, the effects of attaching stationary coaxial wires in the outer cavity region should be studied. This is expected to enhance the radial velocity distribution within the whole packaging and hence break down the apparent shear stress which is responsible for gas maldistribution phenomena. Also, attaching inner coaxial wires to the inner cavity region can destroy the tangential velocity component of the gas feed and thereby mitigate the effects of the free vortex flow in this region.

### Supplementary Information

Supplementary Information 1.

Supplementary Information 2.

## Data Availability

The data generated or analyzed during this study are included in supplementary information files and other datasets used and/or analyzed during the current study are also available from the corresponding author on reasonable request.

## List of symbols

$$P$$: Pressure, Pa
$P_{k}$: Rate of production of turbulence, m^2^/s^3^
U, V: Velocity, m/s
$$a_{p}$$: Packing density, m^2^/m^3^
$$dA$$: Differential area, m^2^
$$dV$$: Differential volume, m^3^
g: Gravity acceleration, m/s^2^
$h$: Packing axial height, m
$k$, TKE: Turbulent kinetic energy, m^2^/s^2^
$$r$$: Radius, m
u, v: Velocity, m/s
$\overline{u}$: Timed-averaged velocity, m/s
$$\overline{{u^{\prime}}}$$: Fluctuated velocity, m/s
rpm: Revolution per minute

## Greek letters

µ: Dynamic viscosity, Pa. s
$$\nu$$: Kinematic viscosity, m^2^/s
$\sigma_{k}$: Turbulence kinetic energy Schmidt number, [-]
$\sigma_{\varepsilon }$: Turbulence dissipation rate Schmidt number, [-]
$\tau$: Shear stress, Pa
ρ: Density, kg/m^3^
ω: Angular velocity, rad/s

## Subscripts

$$av$$: Average
$$app$$: Apparent
c: Casing wall
i: Inner edge
o: Outer edge
$$r$$: Radial component
w: Wetted
$$\theta$$: Tangential component
