# Supplementary material for: CFD microscale modelling of flow behavior in different parts of a rotating packed bed
Source: Sci Rep. 2023 Dec 16;13:22419. doi: 10.1038/s41598-023-49905-5 (PMC10725488; doi:10.1038/s41598-023-49905-5)
Supplement: Supplementary file 1 — Supplementary Information 1. [file 41598_2023_49905_MOESM1_ESM.docx]

**Appendix**

**Approximate estimating of the uncertainty** $\left( \boldsymbol{E} \right)$ **related to each pressure gradient data point utilizing Richardson extrapolation at an operating condition of 400 rpm and 30 m^3^/h.**

| $\frac{\boldsymbol{\Delta}\boldsymbol{P}}{\boldsymbol{\Delta r}}\left( \boldsymbol{h} \right)$ | $\frac{\boldsymbol{\Delta}\boldsymbol{P}}{\boldsymbol{\Delta r}}\left( \boldsymbol{2}\boldsymbol{h} \right)$ | $\boldsymbol{\rho}\frac{\boldsymbol{V}_{\boldsymbol{Slip-\theta}}^{\boldsymbol{2}}}{\boldsymbol{r}}$ | $\boldsymbol{E\cong}\frac{\boldsymbol{4}}{\boldsymbol{3}}\left( \frac{\boldsymbol{\Delta}\boldsymbol{P}}{\boldsymbol{\Delta r}}\left( \boldsymbol{2}\boldsymbol{h} \right)\boldsymbol{-}\frac{\boldsymbol{\Delta}\boldsymbol{P}}{\boldsymbol{\Delta r}}\left( \boldsymbol{h} \right) \right)$ |
| --- | --- | --- | --- |
| 369.5 | 377.5 | 349.9825 | 10.66667 |
| 449 | 478.65 | 437.4475 | 39.53333 |
| 581.9 | 582 | 526.75 | 0.133333 |
| 666.1 | 666 | 627.3225 | 0.133333 |
| 766.6 | 767 | 748.475 | 0.533333 |
| 934.3 | 972.5 | 898.5375 | 50.93333 |
| 1148 | 1165 | 1078.98 | 22.66667 |
| 1396 | 1401 | 1262.975 | 6.666667 |
