# Supplementary material for: CFD microscale modelling of flow behavior in different parts of a rotating packed bed
Source: Sci Rep. 2023 Dec 16;13:22419. doi: 10.1038/s41598-023-49905-5 (PMC10725488; doi:10.1038/s41598-023-49905-5)
Supplement: Supplementary file 2 — Supplementary Information 2. [file 41598_2023_49905_MOESM2_ESM.docx]

Supplementary materials

**CFD Microscale Modelling of RPB - Data Report (DOI://zenodo.org/uploads/10161257).**
